# Detection of Interpretable and Fine-Grained Brain Tumor Magnetic Resonance Imaging Based on Progressive Pruning: Machine Learning Model Development and Validation Study

**DOI:** 10.2196/84095

**Published:** 2026-04-29

**Authors:** Yupeng Liu, Shuwei Song, Shibo Lian, Xiaochen Zhang

**Affiliations:** 1 School of Computer Science and Technology, Harbin University of Science and Technology, Harbin, China; 2 Heilongjiang Institute of Technology, No. 999 Hongqi Street, Daowai District, Harbin City, Heilong, Harbin, 150001, China, 86 13608701118

**Keywords:** magnetic resonance imaging, MRI, brain tumor, convolution Prewitt-and-pooling–based preprocessing, progressive hybrid pruning strategy, dynamic convolution-based C3k2, feature fusion, medical imaging, brain tumor detection, deep learning, class activation mapping, Eigen-CAM, lightweight model

## Abstract

**Background:**

Brain tumor is one of the most malignant diseases of the central nervous system, and early accurate detection is of great significance for improving patient survival rate. However, the heterogeneity of brain tumors in terms of morphology, size, and location on magnetic resonance imaging (MRI) image, as well as their similarity to surrounding normal brain tissue, poses significant challenges for tumor detection.

**Objective:**

This study aims to develop a high-performance brain tumor detection framework that integrates feature enhancement, channel attention, and progressive pruning, achieving an optimal balance between detection accuracy, model efficiency, and interpretability for slice-level MRI tumor localization tasks.

**Methods:**

This paper proposes a convolution Prewitt-and-pooling–based preprocessing (CSPP) approach, based on the “you only look once” version 11 (YOLOv11) framework, which highlights important structural detail more effectively than traditional statistics. A dynamic convolution–based C3k2 (DCC) module was integrated to more efficiently capture both local and global features. A channel prior convolutional attention (CPCA) module was introduced before the detection head, enabling the network to specifically focus on information-rich channels and key spatial regions. Through a progressive hybrid pruning strategy (PHPS), the model was optimized for efficient inference. Furthermore, Eigen-class activation mapping (Eigen-CAM) was used to interpret the prediction result, making them more transparent.

**Results:**

Extensive experiments on 3 brain tumor MRI datasets demonstrated the superior performance of CDCP-YOLO (CSPP-DCC-CPCA-PHPS–YOLO). On Br35H, the mean average precision (mAP) at an intersection-over-union (IoU) threshold of 0.5 (mAP_0.5_) increased by 2.6%, average mAP over several IoU thresholds (0.50-0.95; mAP_0.5:0.95_) increased by 5.9%, and number of floating-point operations (×10⁹; GFLOPs) decreased by 47.7%. On Roboflow, mAP_0.5_ increased by 19.5%, mAP_0.5:0.95_ increased by 7.7%, and GFLOPs decreased by 47.7%. On Capstone, mAP_0.5_ increased by 6.9%, mAP_0.5:0.95_ increased by 5.8%, and GFLOPs decreased by 47.7%.

**Conclusions:**

The proposed CDCP-YOLO framework achieves an optimal balance between accuracy, efficiency, and interpretability, providing a lightweight and reliable solution for slice-level brain tumor detection in MRI images.

## Introduction

### Background

Brain tumors are a type of highly complex and rapidly progressing major disease within the central nervous system. Their malignancy is often characterized by extreme invasiveness and recurrence, posing a serious threat to patients’ lives and health. Medical research indicates that accurate identification and timely diagnosis of tumor types in the early stages can significantly extend patient survival and improve quality of life. Magnetic resonance imaging (MRI) has become the gold standard for preoperative imaging diagnosis of most brain tumors due to its noninvasive, high-resolution, and multimodal imaging advantages, playing a key role in the auxiliary diagnosis and efficacy evaluation of brain tumors [1-3]. Although MRI provides rich information at the image level, brain tumor detection still faces several challenges:

1. The tumor shape is complex and diverse, with blurred boundaries, and its grayscale distribution often overlaps with normal brain tissue, making it difficult to distinguish and locate accurately.
2. The anatomical structure of the brain is complex and varies significantly between individuals, making it difficult for traditional methods to establish a universally applicable and robust expression model.
3. Manual image segmentation is not only time-consuming and labor-intensive but also susceptible to subjective factors, necessitating efficient and intelligent auxiliary diagnostic tools.

In recent years, an increasing number of researchers have begun to focus on intelligent methods for brain tumor detection. The main research is based on digital image processing and machine learning to assist in identifying brain tumor regions [4]. This type of method usually first uses MRI image acquisition to obtain brain image data; then uses image processing to extract key visual features, such as gray scale, shape contours, and texture patterns; and finally inputs these features into support vector machines, random forests, or k-nearest neighbors for judging the tumor site and type. This type of method, which relies on handcrafted features, is effective for the detection of target lesions, but when faced with complex organizational structures and highly heterogeneous tumor morphologies, it often exhibits poor robustness, sensitivity to image noise, and difficulty in generalization, which limits its widespread application in clinical practice. With the rapid development of the convolutional neural network (CNN), target detection has entered a new stage with end-to-end learning as its core. Girshick et al [5] proposed region-based CNN (R-CNN), which first extracts candidate target regions and then uses support vector machines for classification, significantly improving detection performance. Subsequently, Fast R-CNN and Faster R-CNN [6,7] further accelerated detection speed and improved accuracy by sharing features and a region proposal network. To achieve real-time detection, Redmon et al [8] proposed the “you only look once” (YOLO) series of algorithms, which transformed target detection into a regression problem, simultaneously predicting multiple bounding boxes and categories in a single forward pass, greatly increasing the detection speed. Subsequent versions of YOLO have continuously improved the detection accuracy and network structure and are widely used in scenarios such as autonomous driving and medical image analysis. In addition, single shot multibox detector (SSD) [9] also achieves a balance between speed and accuracy by detecting at multiple feature scales. These deep learning–based algorithms not only overcome the limitations of traditional manual feature engineering but also greatly improve the modeling accuracy and practical application value, becoming the mainstream direction of current image target detection, including brain tumor detection.

### Related Works

#### YOLO-Based Detection on MRI

In recent years, target detection models based on YOLO have been widely applied and continuously optimized in medical imaging. In the area of noninvasive disease detection, researchers have primarily focused on introducing attention mechanism, multitask learning, and lightweight model optimization. Chen et al [10] proposed the MSA-YOLOv5, which combines multiple attention mechanisms to focus on the automatic detection of lesions in acute ischemic stroke. In a multimodal MRI model, the detection of small lesions and embolic signals is enhanced, improving detection accuracy while reducing the number of parameters. Tang et al [11] developed a YOLOv5 model based on the squeeze-and-excitation attention mechanism, which improved the detection of Parkinson disease. The model enhances detection accuracy by adaptively focusing on key features, especially in distinguishing substantia nigra and red nucleus lesions in T2-weighted MRI. Wang et al [12] introduced an improved YOLOv5 architecture for the diagnosis and grading of lumbar disc herniation. This method adds an attention module to the Cross Stage Partial part and enhances the Spatial Pyramid Pooling-Fast part, achieving multitask learning for both classification and grading of Pfirrmann grades, highlighting prominent features in the intervertebral space and high-intensity zones.

In the context of brain tumor MRI detection, the research on improving YOLO has also advanced rapidly. Kang et al [13] proposed a new YOLO architecture named RCS-YOLO, which is optimized for brain tumor detection in medical imaging. RCS-YOLO uses reparameterized convolution combined with channel shuffle (RCS) to enhance the model’s computational efficiency and detection accuracy. Kang et al [14] developed BGF-YOLO, an improved YOLOv8 that enhances brain tumor MRI detection performance by introducing multilevel feature fusion, a dynamic attention mechanism, and an additional detection head. Kang et al [15] proposed a new YOLO architecture, pretrained knowledge-guided YOLO (PK-YOLO), optimized for brain tumor detection in multiplanar MRI slices. PK-YOLO uses a pretrained RepViT backbone network combined with sparse mask modeling technology and Focaler-intersection-over-union (IoU) regression loss to improve the detection performance of small targets, making it the first YOLO object detector to introduce pretrained knowledge guidance. Dixit et al [16] proposed a brain tumor detection method based on YOLOv4-tiny, which uses transfer learning and a fine-tuning technique optimized for MRI images. This method leverages features from the pretrained Common Objects in Context (COCO) dataset with the 29-layer YOLOv4-tiny architecture, improving the model’s computational efficiency and detection accuracy through precise hyperparameter tuning. Abdusalomov et al [17] proposed an improved YOLOv7 architecture optimized for brain tumor detection in MRI images. This method integrates a Convolutional Block Attention Module, a Spatial Pyramid Pooling Fast+ layer, and a Bidirectional Feature Pyramid Network to improve the detection accuracy for glioma, meningioma, and pituitary tumors.

#### Pruning Method

As the complexity of the deep neural network continues to increase, how to reduce computational and storage requirements while maintaining high performance has become a key challenge in medical imaging processing. Pruning techniques offer an effective solution to this problem and have demonstrated considerable potential in various medical image analysis applications. Fernandes et al [18] proposed a generative adversarial pruning method based on an evolutionary strategy, specifically optimizing medical image diagnosis. This method selects options through minimal Wasserstein distance. Wu et al [19] developed the FairPrune method, a new technique that achieves fairness by pruning, specifically applied to the diagnosis of skin diseases. This method prunes based on differences in parameter importance, significantly improving the fairness of the model across different demographic groups. Adnan et al [20] proposed a structured pruning method specifically for optimizing the U-Net architecture. This method addresses the pruning complexity between the encoder and decoder in U-Net, compressing the model by assessing the importance of individual channels and tasks. Fernandes et al [21] further developed a structured pruning framework that integrates multitask learning and pruning. This method uses iterative pruning and block-based network deepening, optimizing the model with a policy-based and multiobjective decision-making process. Cocosco et al [22] proposed a fully automated and nonparametric brain tissue classification method. This method uses a nonparametric implementation, training sample selection through the minimum spanning tree method and stereotaxic space priors, showing significant performance improvement in subjects with large morphological variations. Xuan et al [23] introduced a pruning method for k-space subsampling and reconstruction based on a generative model. This method is inspired by network pruning, starting with a fully sampled k-space model and iteratively removing less important k-space phase encoding, demonstrating good performance in single-coil and multicoil MRI reconstruction. Graziani et al [24] developed an interpretable pruning strategy on CNN specifically for scale-variant features in medical images. This method uses deep learning interpretability techniques to analyze the hierarchical scale coding of the InceptionV3 and ResNet50 architectures, finding that scale information peaks in the middle layers and decreases near the softmax layer. This discovery leads to a pruning strategy that significantly improves the performance of nucleus regression and mitosis classification in histopathological images. Holste et al [25] systematically analyzed the impact of the pruning on medical image classification across various long-tailed multilabel disease datasets for the first time. This study in the chest X-ray image diagnosis experimentally demonstrated that the pruning has differential effects on different diseases, with rare diseases being more susceptible to being “forgotten” than common diseases. The study also introduces the concept of pruning-identified exemplars, revealing through human reader studies that pruning-identified exemplars often have more label noise, lower image quality, and higher diagnostic uncertainty. Saleh et al [26] investigated the effectiveness of different network architectures (GoogLeNet, ResNet, and EfficientNet) combined with transfer learning and the network pruning algorithm for medical image classification. They validated the effectiveness of these techniques in both brain tumor classification and chest X-ray inflammation detection. Jaiswal et al [27] proposed a pruning-assisted, self-supervised image localization method. This method uses a “learning by forgetting” training scheme, which significantly improves skin disease localization performance under unsupervised, weakly supervised, and sparsely supervised settings.

#### Visualization Based on Class Activation Mapping

In recent years, visualization methods based on class activation mapping (CAM) have been widely applied in interpretability research on deep neural networks. The classic gradient-weighted CAM (Grad-CAM) [28] calculated specific category information relative to the feature maps of each layer by determining the weights of each channel, thereby generating a heat map that is closely related to the target category. However, when dealing with multitarget and complex background scenes, Grad-CAM sometimes struggles to capture fine-grained details and local features. To address this limitation, Grad-CAM++ [29] further improved the high-order information, significantly enhancing sensitivity to small targets and overlapping regions. On the other hand, Eigen-CAM [30], which got rid of the reliance on gradient calculation, was based on principal component analysis to extract convolution features, generating class-independent visual interpretations in an unsupervised manner. Furthermore, to further enhance the detail and perceptual awareness of heat maps, LayerCAM [31] was proposed to fuse the activation maps from the deep and shallow convolution layers, resulting in a more pixel-level local correspondence and generating finer-grained heat maps that better express boundary and structural information. To validate the CAM method in medical diagnosis, many recent studies have explored its use. Windisch et al [32] proposed a ResNet50-based brain tumor detection model and used Grad-CAM for model interpretability analysis, used for the identification of meningiomas and gliomas in MRI slices. Shawon et al [33] proposed a cost-sensitive deep neural network that integrated multiple interpretable techniques (including Grad-CAM, LIME, and Score-CAM) for model interpretation, used for brain tumor detection under imbalanced data conditions. Dasanayaka et al [34] proposed a deep learning model based on U-Net and DenseNet, which used Grad-CAM to generate heat maps for brain tumor segmentation and classification. This combined strategy not only achieves effective segmentation and classification but also provides effective visualization. Zeineldin et al [35] proposed an enhanced EfficientNetv2 that integrates a global attention mechanism and efficient channel attention and uses Grad-CAM visualization for model interpretation, applied to MRI-based brain tumor classification. Guluwadi et al [36] proposed a brain MRI detection method that combines ResNet50 with Grad-CAM.

### Objectives

In recent years, the rapid advancement of deep learning has significantly propelled the development of brain tumor detection, achieving remarkable results. However, current brain tumor detection models still face several challenges:

1. The boundaries of brain tumors are often blurry and the structures are irregular. The shallow features extracted by traditional convolutions are insufficient to effectively capture the key regions, leading to high rates of missed detection and false positives.
2. The parameter size of the target detection model is massive, and the computational overhead is high, which is not conducive to deployment on clinical edge devices, thus limiting their practicality. Traditional pruning methods, typically based on a single-weight threshold for coarse-grained pruning, can easily damage key model structures. At the same time, the sparse fine-tuning process, due to its inefficiency, struggles to meet the demands of high efficiency and deployability in medical scenarios.
3. Most target detection methods lack interpretability, making it difficult to clearly present the decision-making basis of the model to doctors, which limits its credibility in auxiliary clinical decision-making.

This paper proposes an interpretable, fine-grained brain tumor MRI detection method based on progressive pruning to address these challenges in an integrated manner. Importantly, CDCP-YOLO (CDC-YOLO with PHPS) is not a simple stacking of existing techniques but a task-driven co-design framework specifically tailored to the structural complexity, computational constraints, and interpretability requirements of brain tumor MRI detection. Its methodological distinctions are summarized as follows. First, unlike RCS-YOLO, which primarily accelerates inference through convolution reparameterization and channel shuffling, CDCP-YOLO introduces structure-aware input-level modeling (convolution Prewitt-and-pooling–based preprocessing [CSPP]) that explicitly embeds classical edge priors into the network. This design targets blurred and ambiguous tumor boundaries at the earliest stage of feature extraction—an issue that reparameterization alone cannot effectively address. Second, whereas BGF-YOLO improves detection performance mainly through multilevel feature fusion and additional detection heads, CDCP-YOLO adopts dynamic convolution–based C3k2 (DCC) feature adaptation, enabling input-dependent kernel generation. This strategy allows the network to flexibly adapt to heterogeneous tumor morphologies and scales without introducing detection head redundancy. Third, while PK-YOLO relies on external pretrained knowledge and sparse mask modeling to enhance small-target detection, CDCP-YOLO does not depend on any external pretraining paradigm. Instead, it improves representation capacity through internal, data-adaptive mechanisms, ensuring robustness and generalizability under limited or domain-specific medical datasets. Fourth, existing pruning-based detectors typically use single-criterion or one-shot pruning strategies that may disrupt critical feature pathways. In contrast, the proposed progressive hybrid pruning strategy (PHPS) jointly considers global channel sparsity and local structural dependency in a staged manner, enabling aggressive model compression while preserving detection-critical structures. Finally, unlike prior works that treat Grad-CAM–style visualization as a post hoc analysis tool, CDCP-YOLO tightly integrates Eigen-CAM into the detection head, treating interpretability as a core design objective rather than an auxiliary component. This integration ensures semantic consistency between detection results and visual explanations, which is essential for clinical decision support.

The main contributions of this work are summarized as follows:

1. Multiscale feature enhancement mechanism: a CSPP module was introduced at the model’s input stage, integrating Prewitt edge detection with pooling operations to strengthen the construction of tumor edges and structures. A DCC module was introduced into the backbone and neck networks to achieve adaptive expression for different tumor forms and sizes. A channel prior convolutional attention (CPCA) module was introduced before the detection head to guide the network to focus on the key region of the most discriminative brain tumor.
2. Lightweight pruning strategy: a progressive hybrid pruning strategy was proposed, combining L1-norm and GroupNorm feature statistics. The pruning process is carried out in stages, prioritizing the pruning of redundant channels while maximally preserving key information flow. This method significantly reduces parameter size and computational overhead while effectively mitigating the performance degradation caused by large-scale pruning.
3. Interpretable confidence enhancement: a gradient-free principal component analysis method was adopted, which can generate clear saliency heat maps, visually demonstrating the most important basis for the brain tumor MRI model’s decision-making. This significantly improves the transparency, credibility, and practicality of the model in clinical applications.

## Methods

### Overall Framework

**Figure 1.**
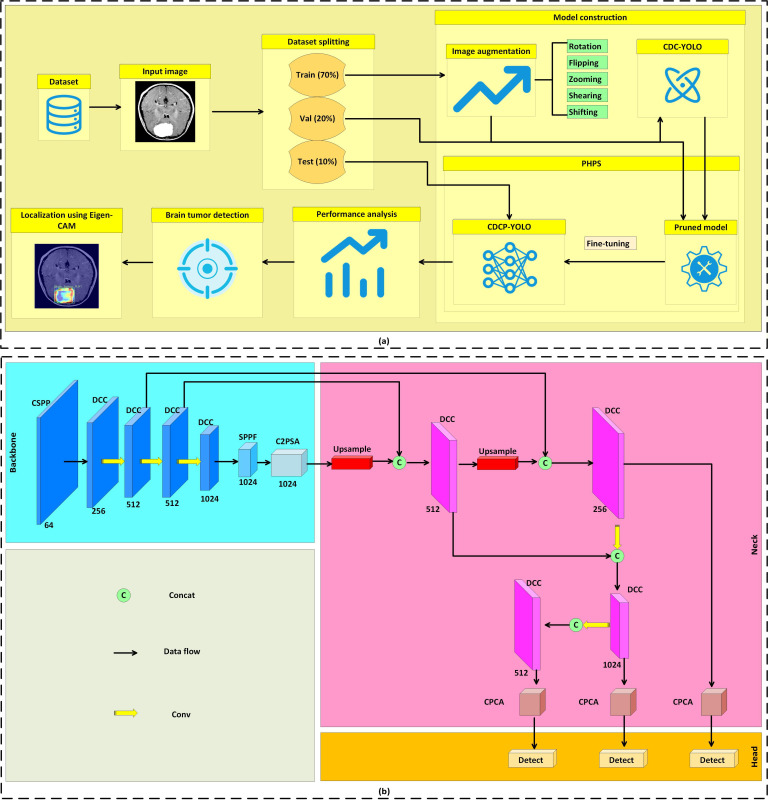
Overall framework and network architecture of the proposed CDCP-YOLO model for brain tumor magnetic resonance imaging detection. (A) The complete training and pruning pipeline, including feature enhancement, progressive hybrid pruning, and fine-tuning. (B) Detailed architecture of the CDC-YOLO backbone, neck, and detection head, where CSPP enhances edge features at the input stage, DCC adapts to multiscale tumor morphology, and channel prior convolutional attention (CPCA) guides the detection head to focus on discriminative channels and spatial regions. C2PSA, C2 block integrated with partial self-attention; CAM, class activation mapping; CDC-YOLO, cross-scale dynamic convolution–based YOLO; CDCP-YOLO, CDC-YOLO with PHPS; CSPP, convolution Prewitt-and-pooling–based preprocessing; Concat, concatenation; Conv, convolution; DCC, dynamic convolution–based C3k2 module; SPPF, spatial pyramid pooling-fast; Val, validation subset; YOLO, “you only look once” framework.

This paper proposes an interpretable and fine-grained brain tumor detection model based on progressive pruning. As shown in Figure 1A, the overall framework was as follows: MRI images were trained on the improved CDC-YOLO network, and an efficient CDCP-YOLO model was obtained through a progressive hybrid pruning strategy and fine-tuning. As shown in Figure 1B, CDC-YOLO is composed of three main structures:

1. Backbone stage: the CSPP module is used to replace the first 2 convolutional layers. CSPP combines Prewitt edge detection with pooling operations to enhance the model’s initial feature extraction capability for edges and textures. Subsequently, the main network uses multiple DCC modules, using dynamic convolution to enhance the perceptual ability for multiscale tumor regions.
2. Neck stage: multiscale feature maps are fused through upsampling and concatenation. The C3k2 module introduces dynamic convolution and key location fusion, and the CPCA module is introduced, combining channel and spatial attention mechanisms to guide the model to focus on key diagnostic areas and improve detection accuracy.
3. Head stage: multiple detection heads of different sizes are used to detect targets of different sizes.

### Convolution Prewitt-and-Pooling–Based Preprocessing

**Figure 2.**
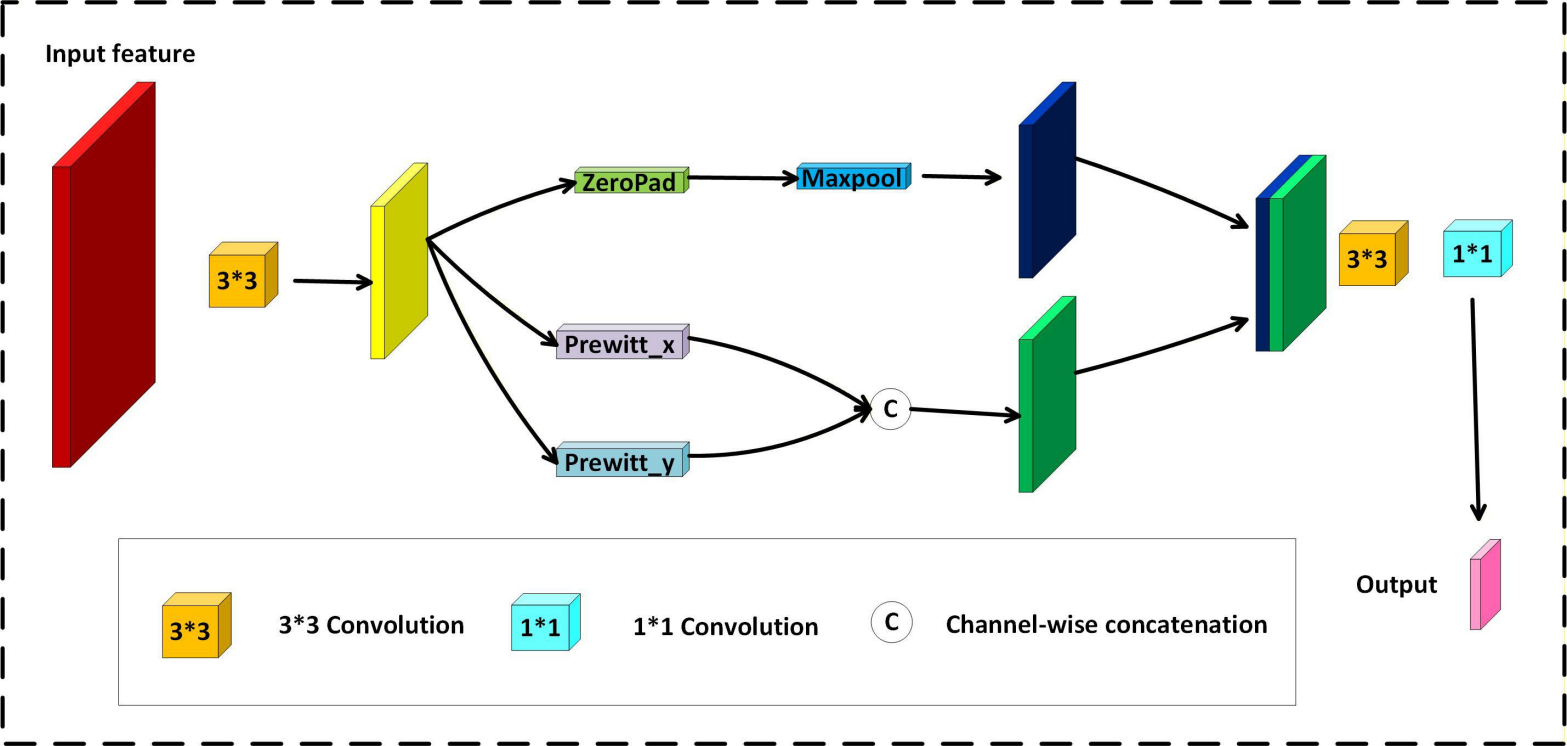
Structural illustration of the convolution Prewitt-and-pooling–based preprocessing (CSPP) module. The module integrates standard convolution, Prewitt edge detection (horizontal and vertical), and max pooling to enhance tumor boundary and texture representation at the input stage. Maxpool, maximum pool.

The head of YOLOv11 includes 2 initial convolution layers, primarily used to extract initial features from the input image. By extracting deeper features layer by layer, the network can construct sufficient information for effective target detection. To enhance the model’s perceptual ability for edge structures, the first 2 standard convolutional layers in YOLOv11 are replaced with a self-designed CSPP (Figure 2), which more effectively extracts contour information and local texture features from the image. As the input feature processing unit of the entire CDCP-YOLO, CSPP integrates standard convolution, Prewitt edge detection, and multiscale pooling operations, aiming to enhance the model’s perceptual ability for brain tumor edge features and its adaptability to different shapes.

First, the input image X undergoes a 3×3 convolution operation to obtain the initial feature map $X_{1}$; this operation completes spatial compression and channel expansion.


(1) $$\begin{array}{c} X_{1}=Conv\left(X\right)\; \end{array}$$

Next, the Prewitt edge detection operator is applied to $X_{1}$, filtering in the horizontal direction $G_{x}$ and the vertical direction $G_{y}$ to extract the edge response in the image.


(2) $$\begin{array}{c} X_{Prewitt}=PrewittConv\left(X_{1}\right) \end{array}$$

The output of the Prewitt operator is obtained by calculating the Euclidean distance to combine the responses from both directions, resulting in the complete edge information $X_{Prewitt}$.


(3) $$\begin{array}{c} X_{Prewitt}=\sqrt{{\left(X_{1}\times G_{x}\right)}^{2}+{\left(X_{1}\times G_{y}\right)}^{2}} \end{array}$$

Simultaneously, another branch inputs $X_{1}$ into a max-pooling layer, and the spatial dimensions are kept unchanged through zero-padding, generating the pooled feature $X_{pool}$. The 2 output features, $X_{Prewitt}$ and $X_{pool}$, are concatenated along the channel dimension to generate the feature $X_{2}$. This fusion retains both structural edges and spatial context information.


(4) $$\begin{array}{c} X_{2}=Concat\left(X_{Prewitt},X_{pool}\right) \end{array}$$

The fused feature map $X_{2}$ is then passed through a 3×3 convolution to further extract high-level features.


(5) $$\begin{array}{c} X_{3}=Conv_{3\times 3}\left(X_{2}\right) \end{array}$$

Finally, the number of channels is compressed, and the dimensions are adjusted using a 1×1 convolution to generate the final feature Y, which serves as the input to the backbone structure.


(6) $$\begin{array}{c} Y=Conv_{1\times 1}\left(X_{3}\right) \end{array}$$

CSPP uses the Prewitt operator in both horizontal and vertical directions to automatically extract prominent edge contours in the image, significantly enhancing the detection capability for blurry contours and unclear tumor regions. At the same time, the output of Prewitt is fused with the original feature map, which not only preserves the local details but also strengthens the expression of structural information. In addition, the pooling operation compresses the spatial dimensions while keeping the feature map size unchanged, providing the network more robustness to variations in tumor size and location.

The CSPP module is designed to introduce deterministic structural priors into the early stages of feature extraction. While standard convolutional layers learn kernels stochastically, the inclusion of a fixed Prewitt operator provides explicit edge-sensitive cues that are critical for delineating tumor boundaries. We acknowledge that in medical imaging physics, difference operators are typically sensitive to high-frequency noise. Therefore, rather than using the Prewitt output directly, the CSPP module integrates it through a multistage fusion strategy. Specifically, the gradient maps are processed via max pooling to perform local maximum selection, which emphasizes strong structural edges while suppressing isolated noise spikes. These features are then fused with learnable convolutional features using a 1×1 convolution layer and batch normalization. This design allows the network to adaptively weight the explicit structural priors against learnable representations, ensuring that the model captures fine-grained morphological details without amplifying imaging artifacts.

### Dynamic Convolution-Based C3k2

The C3k2 module is a core feature extraction unit in YOLOv11, and its design goal is to achieve the best balance between feature representation capability and computational efficiency. This module uses a multibranch structure and residual connections to enhance the multiscale feature extraction capability while maintaining training stability. C3k2 supports two configuration modes:

1. In the C3k=True mode, a lightweight C3k branch is used. The input features are divided into multiple groups for separate processing and then fused through concatenation. This group-wise convolution strategy significantly reduces computational complexity, making it particularly suitable for applications with high real-time requirements.
2. In the C3k=False mode, a more complex bottleneck structure is introduced, combined with additional convolution and activation layers to extract deeper and more discriminative features, thus showing superiority in more accuracy-sensitive tasks.

**Figure 3.**
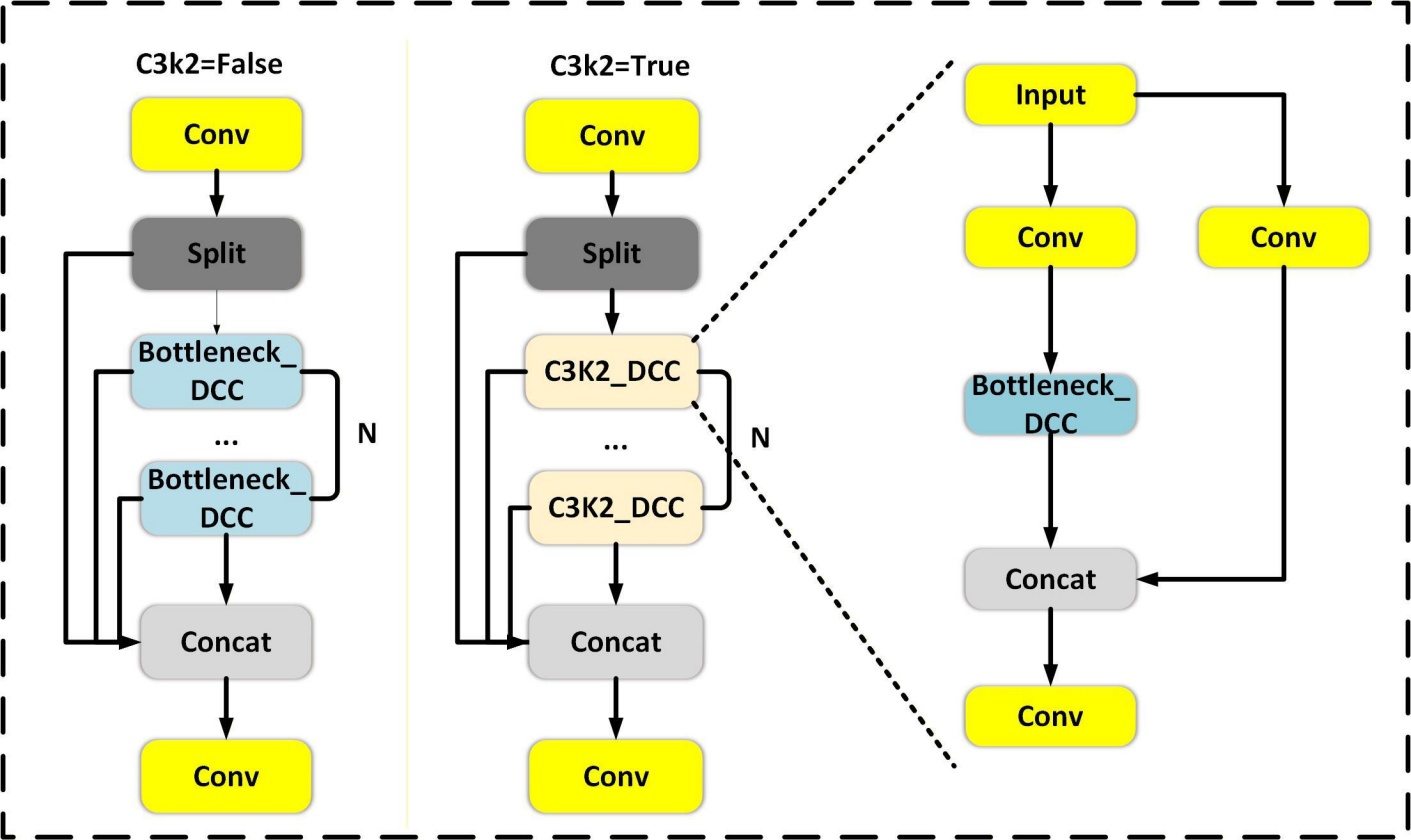
Architecture of the dynamic convolution–based C3k2 (DCC) module. The convolution kernel is dynamically generated according to the global context of the input feature map, enabling adaptive modeling of tumors with diverse sizes and shapes. Concat, concatenation; Conv, convolution.

To enhance the network’s modeling capability for complex tumor regions, in this study, we built a DCC module (Figure 3) based on the original C3k2 module by introducing a dynamic convolution [37]. The DCC module enhances the model’s capabilities in multiscale structure modeling, detail preservation, and contextual awareness by replacing the fixed convolution with the input-adaptive dynamic convolution.

Given an input feature $X\in R^{C_{in}\times H\times W}$, the DCC module uses a dynamic kernel *W(X*) to perform the convolution operation:


(7) $$\begin{array}{c} Y=W\left(X\right)\times X \end{array}$$

The generation process of the convolution kernel $W\left(X\right)$ depends on the global context information of the input image. To achieve this weight regeneration, a global average pooling layer is first used to compute the channel-wise global vector:


(8) $$\begin{array}{c} g=\frac{1}{H\times W}\sum_{i=1}^{H}\sum_{j=1}^{W}X\left[:,i,j\right] \end{array}$$

Then, 2 fully connected layers and a nonlinear activation function are used to generate the dynamic attention weights:


(9) $$\begin{array}{c} \theta =FC_{2}\left(\sigma \left(FC_{1}\left(g\right)\right)\right) \end{array}$$

Here *σ*() is the ReLU activation function, and ${FC}_{1}$ and ${FC}_{2}$ are the first and second fully connected layers, respectively. The final parameter θ determines the weighting of the dynamic convolution kernel $W\left(X\right)$, thereby adapting to the feature distribution of the current input image for optimal perception.

The C3k2 module mainly extracts higher-level features through multiple bottleneck and convolution layers. This module stacks multiple bottleneck units, replacing standard static convolution operations, enabling the network to adaptively select more suitable feature representations at different spatial scales, and thereby improving the modeling capability for diverse tumor features. Unlike fixed convolution kernels, the dynamic convolution mechanism automatically adjusts the kernel weights based on the input features, making it adaptable to different image contexts. Especially when dealing with tumor regions with blurry boundaries and irregular shapes, dynamic convolution can more finely capture local structures and texture changes. In addition, dynamic convolution also enhances the model’s ability to resist interference from complex backgrounds and low signal-to-noise ratios, reducing the occurrence of missed and false detections. At the same time, the DCC module introduces an input-dependent kernel selection strategy, which endows the C3k2 module with stronger language modeling and morphological adaptation capabilities, providing richer and more discriminative feature representations for subsequent detection heads.

### Channel Prior Convolutional Attention

**Figure 4.**
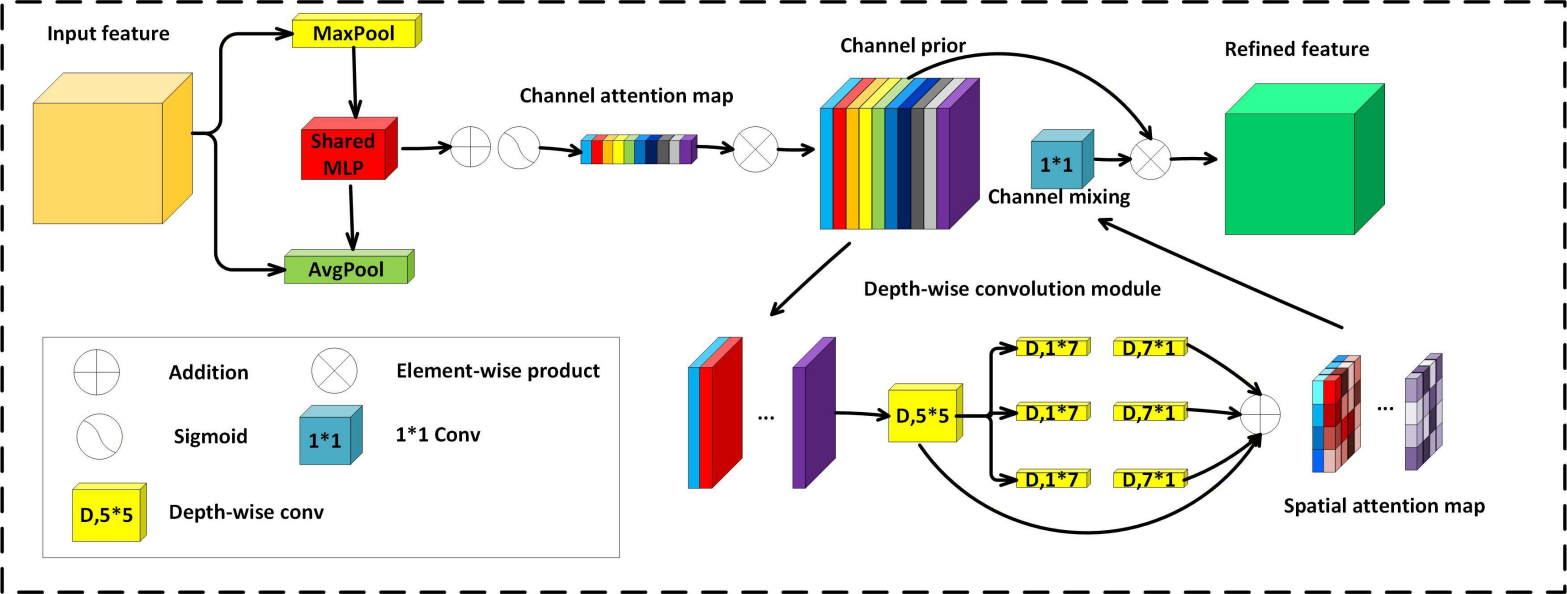
Structure of the channel prior convolutional attention (CPCA) module, which sequentially applies channel attention and spatial attention to emphasize tumor-relevant channels and spatial regions before the detection head. Conv, convolution; MaxPool, maximum pool; AvgPool, average pool.

The detection head is the key part for the final bounding box regression. However, the detection head relies on the feature maps from the previous layer, and these feature maps may contain a large amount of information, where many channels may be redundant or irrelevant to the target detection. The attention mechanism is proposed to focus on important information. The CPCA module (Figure 4) [38] uses multiscale depth-wise separable convolutions to maintain the channel prior while extracting spatial relationships, enabling the network to focus on information-rich channels and key spatial regions. The CPCA module includes the sequential placement of channel attention and spatial attention. The spatial information of the feature maps is aggregated by channel attention through operations such as average pooling and max pooling. The spatial attention is then processed through a shared multilayer perceptron and added to generate a channel attention map. The channel prior is obtained by element-wise multiplication of the input feature and the channel attention map. Subsequently, the channel prior is input into a depth-wise convolution block to generate a spatial attention map. The convolution block receives the spatial attention map to perform channel mixing. Finally, the channel mixing result is element-wise multiplied with the channel prior to obtain the optimized feature as output. The channel mixing process helps to enhance the feature representation.

Given an intermediate feature map $F\in R^{C\times H\times W}$, a 1D channel attention map $M_{c}\in R^{C\times 1\times 1}$ is first inferred through the channel attention module. Then, $M_{c}$ is element-wise multiplied with the input feature F to obtain the channel attention–optimized feature map $F_{c}\in R^{C\times H\times W}$. Subsequently, the spatial attention (SA) processes $F_{c}$ to generate a 3D spatial attention map $M_{s}\in R^{C\times H\times W}$. The final output feature map $\hat{F}$ is obtained by element-wise multiplication of $M_{s}$and $F_{c}$:


(10) $$\begin{array}{c} F_{c}=CA\left(F\right)⊗F \end{array}$$

(11) $$\hat{F}=SA(F_{c})⊗F_{c}$$

, where ⊗ represents element-wise multiplication. Channel attention (CA) is obtained as follows:


(12) $$\begin{array}{c} CA\left(F\right)=\sigma \left(\begin{array}{c} MLP\left(AvgPool\left(F\right)\right)\\ +MLP\left(MaxPool\left(F\right)\right) \end{array}\right) \end{array}$$

, where σ represents the sigmoid function. Spatial attention (SA) is obtained as follows:


(13) $$\begin{array}{c} SA\left(F\right)=Conv_{1\times 1}\left(\sum_{i=0}^{3}Branch_{i}\left(DwConv\left(F\right)\right)\right) \end{array}$$

, where DwConv represents depth-wise convolution, $Branch_{i}$ represents the *i*th branch, and $Branch_{0}$ is an identity connection.

Applying CPCA to the predetection layer optimizes the semantic aggregation capability of the predetection feature layer. The detection head, as the key component for classification and bounding box regression, heavily relies on the quality of the preceding feature layers. However, the original feature maps often contain a large amount of redundant channels or low-response regions, which interfere with the model’s discrimination process, especially in medical images with blurry tumor boundaries and complex backgrounds. CPCA performs adaptive channel modeling, enabling it to automatically identify and enhance significant channels related to tumors, thereby effectively improving the discriminability of features. Furthermore, this mechanism, combined with a spatial attention strategy, guides the network to focus on key areas of the image, enhancing the perceptual ability for fine-grained features such as lesion edges and structural changes. By introducing CPCA before the detection head, the model can perform inference based on more refined, robust, and contextually sensitive features.

### Progressive Hybrid Pruning Strategy

To address the dual requirements of high-precision modeling for complex brain tumor structures and efficient inference, we propose the PHPS, as summarized in Textbox 1. The PHPS first uses an L1-norm–based channel importance evaluation to perform coarse-grained pruning, enabling the rapid removal of globally redundant feature channels. Subsequently, GroupNorm-based grouping information is incorporated to conduct fine-grained structural pruning, which corrects channel importance by considering local structural dependencies and avoids distortions caused by sole reliance on weight magnitude.

Textbox 1. **Pruning processing**

- Input: pretrained model parameters W, overall pruning ratio s, pruning threshold SC, step pruning ratio M.
- Output: pruned and fine-tuned model W1

1.  Initialize CPC-YOLO model.
2.  Set target sparsity s; define thresholds SC.
3.  Initialize current global sparsity S←0.
4.  While S<s, do:
5.    For each prunable layer l in the model, do:
6.  If the current layer’s sparsity SL < SC, then:
7.   Compute importance score: $I_{LI}(w_{i})$.
8.       Sort channels in ascending order.
9.   Prune the lowest-ranked channels with ratio M.
10.   Else:
11.      Compute importance score:$I_{GN}(G_{j})=\sqrt{\sum_{w_{i}\in G_{j}}w_{i}^{2}}$.
12.       Sort groups by $I_{GN}(G_{j})$ in ascending order.
13.       Prune the lowest-ranked groups with ratio M.
14. End if
15.   End for
16.  Update the global sparsity S.
17.  End while
18.  Fine-tune the pruned model on the training dataset.
19.  Return the final pruned model $W_{1}$.

Through this dual evaluation mechanism of global coarse-grained pruning combined with local fine-grained structural correction, the PHPS can more accurately preserve discriminative feature channels under challenging conditions such as blurred tumor boundaries and high intertumor heterogeneity. Importantly, the PHPS adopts a progressive pruning process, in which sparsity is gradually increased toward a predefined global target. Model fine-tuning is performed only once after the entire pruning process is completed, allowing effective recovery of detection performance while avoiding excessive training overhead. This design achieves a balanced optimization between model compression and precision retention.

### Interpretability

In the detection head output stage, the feature map is directly sent to the Eigen-CAM branch while being used for bounding box regression. The most representative convolution activation regions are extracted through principal component analysis. This design not only ensures the semantic consistency between interpretation and detection but also allows for reverse influence on model optimization during training. The extraction of saliency regions enables the network to focus more on real lesion boundaries and key structures, enhancing the model’s adaptability to blurry edges and complex backgrounds. Unlike traditional Grad-CAM, which relies on backpropagation, Eigen-CAM is based on forward features, is class agnostic, lightweight, and efficient, making it more suitable for brain tumor MRI detection.

In this work, Eigen-CAM was applied to the high-level convolutional feature maps before the detection head, rather than to class-specific logits. Although Eigen-CAM is a class-agnostic visualization method that does not rely on gradients with respect to a specific category, the visualized activations are still strongly task driven. Since the detector is trained to localize tumor regions using bounding box regression and objective supervision, the learned high-level features inherently emphasize spatial regions that are most relevant to tumor detection. As a result, the generated heat maps naturally concentrate on tumor areas instead of irrelevant anatomical structures such as the skull or eyes.

For a certain convolution feature layer $F\in R^{C\times H\times W}$, it is first reshaped into a 2D matrix:


(14) $$\begin{array}{c} F^{\prime }\in R^{C\times \left(H\times W\right)} \end{array}$$

Then the covariance matrix is calculated:


(15) $$\Sigma =F^{\prime }F^{\prime^{T}}$$

Next, eigenvalue decomposition is performed on it, and taking the eigenvector $u_{1}$ corresponding to the largest eigenvalue, a heat map is finally generated by weighting the principal components:


(16) $$\begin{array}{c} M=\sum_{c=1}^{C}u_{1}\left[c\right]\cdot F_{c} \end{array}$$

### Ethical Considerations

This study is based entirely on publicly available brain tumor MRI datasets, including Br35H [39], Roboflow dataset [40], and Capstone Brain Tumor dataset [41]. All datasets were obtained from open-access platforms (Kaggle and Roboflow) under their respective terms of use. Each dataset contains anonymized medical images that do not include any personally identifiable information, patient metadata, or clinical identifiers. Therefore, no additional ethical approval or informed consent was required for the use of these datasets.

The datasets were used solely for academic and noncommercial research purposes, strictly following ethical research guidelines and data-sharing policies. All experimental procedures comply with the principles of the Declaration of Helsinki and institutional data protection standards.

## Results

### Experimental Setup

Br35H [39] was used as the main dataset. This dataset is one of the most representative and widely used public benchmarks in brain tumor MRI detection, covering multiple tumor types and MRI modalities. However, it should be noted that the specific clinical metadata regarding the exact MRI sequences (eg, T1-weighted, T2-weighted, or fluid-attenuated inversion recovery) and the specific tumor subtypes are not provided in the original dataset repository. Despite the absence of these specific labels, its high-quality annotations, imaging diversity, and clinical coverage make it a standard for performance evaluation and comparison in automatic brain tumor detection research. To verify the robustness of CDCP-YOLO, the experiments were also conducted on 2 other datasets, Roboflow [40] and Capstone [41]. We strictly adhered to the original dataset split, as detailed in Table 1.

**Table 1.** Dataset splits used for experiments on the Br35H, Roboflow, and Capstone brain tumor magnetic resonance imaging (MRI) datasets.^a^

Dataset	Total number of images, n	Training set, n	Validation set, n	Test set, n
Br35H	801	500	201	100
Roboflow	300	210	60	30
Capstone	911	638	182	91

a The table reports the total number of images and the corresponding training, validation, and test splits for each dataset. These official splits were strictly followed in all experiments to ensure fair evaluation and reproducibility of the reported results.

To enhance the robustness and generalizability of the model, data augmentation was performed during the training phase, including geometric transformations such as rotation, flipping, scaling, and shearing of MRI images, as well as random adjustments to the brightness and contrast. This strategy effectively expands the diversity of the training samples, thereby reducing the risk of model overfitting. This enhancement operation was generated in real time during each training epoch, ensuring that the model obtained “new” samples for training in different epochs, improving its adaptability to complex scenes and diverse tumor morphology. The specific data augmentation hyperparameters used during training included mosaic image composition (mosaic=1.0), geometric transformations (random rotation [degress=10], scaling [scale=0.5], and horizontal flipping [fliplr=0.5]), and color-space perturbations in the hue-saturation-value (HSV) domain (hsv_h=0.015, hsv_s=0.7, and hsv_v=0.4). These augmentation strategies are widely adopted in YOLO-based detection frameworks to enhance data diversity and improve model robustness against variations in object scale, orientation, and imaging conditions.

The experiment was conducted using an Intel Xeon Gold 5320 CPU @ 2.20GHz and an NVIDIA A40 48GB GPU. The training was conducted for 300 epochs, with a batch size of 16, using a stochastic gradient descent (SGD) optimizer, and a patience parameter of 50. The learning rate was set to 0.01. The software environment consisted of Ubuntu 20.04 (Canonical), CUDA 11.8 (NVIDIA), and PyTorch 2.1.0 (Meta AI Research).

After the completion of the initial full training stage, the PHPS was applied to the converged model. Specifically, the base model was first trained for 300 epochs using the standard training configuration described above. Once convergence was achieved, structured pruning was performed according to the PHPS criteria to remove redundant channels and blocks.

Following pruning, the resulting compact model was subjected to a dedicated fine-tuning stage, which was treated as an independent optimization phase and clearly distinguished from the initial training process. During this postpruning fine-tuning, the model was trained for 300 epochs under the same hardware and software environment as the initial training stage, using the SGD optimizer. The batch size was set to 8, the learning rate was fixed at 0.01, and a patience parameter of 50 was applied for early stopping.

### Evaluation Metrics

Precision is the ratio of the number of correctly predicted positive samples to the total number of the detected samples, as shown below:


(17) $$\begin{array}{c} Precision=\frac{T_{P}}{T_{P}+F_{P}}\times 100\% \end{array}$$

Recall is the ratio of the number of correctly predicted positive samples to the number of the actual positive samples, as shown below:


(18) $$Recall=\frac{T_{P}}{T_{P}+F_{N}}\times 100\%$$

Mean average precision (mAP) is the result obtained by averaging the average precision of all categories, used to measure the detection performance of the model across all categories.


(19) $$\begin{array}{c} mAP=\frac{\sum AP}{N_{C}}\times 100\% \end{array}$$

, where mAP_0.5_ represents the mAP at an IoU threshold of 0.5 and mAP_0.5:0.95_ represents the average mAP over several IoU thresholds (0.50-0.95). Params represents parameter size in the model, the number of floating-point operations (×10⁹; GFLOPs) signify computational complexity, and frames per second (FPS) were used to measure inference complexity.

SD was used to measure the variability of a given evaluation metric for the same method across multiple repeated experiments. It was computed as follows:


(20) $$\begin{array}{c} \text{S}\text{D}=\sqrt{\frac{1}{N-1}\sum_{i=1}^{N}{\left(M_{i}-\bar{M}\right)}^{2}\;} \end{array}$$

, where $M_{i}$ denotes the value of the evaluation metric obtained in the ith experiment, and $\bar{M}$ represents the mean value of the corresponding metric over N experiments.

### Comparative Experiments

This paper compares between a variety of mainstream detection models on Br35H, including the classic YOLO series models (such as YOLOv3, YOLOv5n, YOLOv10n, and YOLOv11n); 2-stage detectors (such as Faster R-CNN and Cascade R-CNN); and a 1-stage detection method, TOOD (Table 2). To ensure a fair and unbiased comparison, all baseline models reported in Table 2 were trained from scratch under a unified experimental protocol.

**Table 2.** Performance comparison of different object detection models on the Br35H brain tumor magnetic resonance imaging dataset.^a^

Model	Precision^b^	Recall	mAP_0.5^c^_	mAP_0.5:0.95^d^_	Params (M)^e^	GFLOPs^f^	FPS^g^
YOLOv3-tiny	0.932	0.836	0.898	0.569	12.12	18.9	240.8
YOLOv3	0.926	0.836	0.861	0.602	103.67	282.2	70.2
YOLOv5n	0.891	0.869	0.938	0.601	2.50	7.1	134
YOLOv6n	0.91	0.844	0.931	0.578	4.23	11.8	145
YOLOv8n	0.928	0.843	0.914	0.604	3	8.1	144
TOOD	0.888	0.895	0.925	0.630	32.02	144	13.1
YOLOv10n	0.919	0.84	0.914	0.608	2.27	6.5	131
YOLOv11n	0.904	0.853	0.918	0.585	2.58	6.3	142.2
Faster R-CNN	0.854	0.836	0.896	0.578	41.35	155	15.2
Cascade R-CNN	0.86	0.895	0.874	0.584	69.15	183	14
DINO	0.939	0.75	0.84	0.563	47.54	205	10.5
RCS-YOLO	0.953	0.828	0.824	0.548	45.70	94.5	215.2
BGF-YOLO	0.964	0.885	0.959	0.648	84.30	568.9	32.5
CDC-YOLO	0.881	0.906	0.946	0.660	3.64	7	130
CDCP-YOLO	0.918	0.904	0.944	0.644	2.07	3.3	152

a All models were trained and evaluated under the same experimental protocol to ensure a fair comparison. Higher values indicate better performance for accuracy and speed metrics, whereas lower values indicate better efficiency for model complexity metrics.

b Precision, recall, mAP_0.5_, and mAP_0.5:0.95_ were used to evaluate detection accuracy.

c mAP_0.5_: mean average precision at an intersection-over-union threshold of 0.5.

d mAP_0.5:0.95_: average mean average precision over several intersection-over-union thresholds (0.50-0.95).

e Params (M) was used to measure model size.

f GFLOPs: number of floating-point operations (×10⁹); used to measure computational complexity.

g FPS: frames per second; used to measure inference efficiency.

CDCP-YOLO reached 0.946 in mAP_0.5_, approaching CDC-YOLO’s 0.944, significantly outperforming traditional models such as YOLOv3-tiny (0.898) and YOLOv3 (0.861). At the same time, it achieved a high score of 0.644 on the more challenging mAP_0.5:0.95_ metric, second only to CDC-YOLO (0.660). In addition, CDCP-YOLO’s recall (0.904) and precision (0.918) were both at a leading level, indicating its excellent detection capabilities. In terms of model complexity, the parameter count of CDCP-YOLO was only 2.07M, which is about 1/33 of that of Cascade R-CNN (69.15M) and 1/20 of that of Faster R-CNN (41.35M), and was also smaller than that of YOLOv3 (103.67M), achieving the goal of lightweight design. Its computational complexity was 3.3 GFLOPs, which was also significantly lower than that of YOLOv5n (7.1 GFLOPs) and YOLOv6n (11.8 GFLOPs), demonstrating good computational efficiency. In terms of inference speed, CDCP-YOLO reached 152 FPS, which is significantly faster than Cascade R-CNN (14 FPS), Faster R-CNN (15.2 FPS), and even some lightweight YOLO models such as YOLOv5n (134 FPS) and YOLOv10n (131 FPS), showing significant advantages in practical deployment.

**Figure 5.**
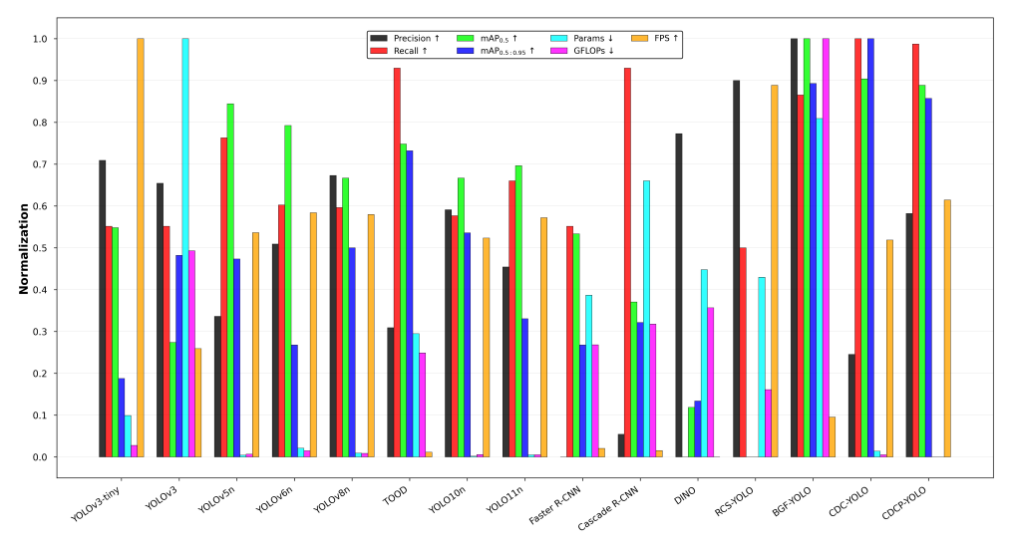
Multimetric normalization comparison of different object detection models on the Br35H brain tumor magnetic resonance imaging dataset. All evaluation metrics are normalized to the range [0, 1] to enable fair comparison across models with different scales. The compared models included YOLOv3-tiny, YOLOv3, YOLOv5n, YOLOv6n, YOLOv8n, YOLOv10n, YOLOv11n, Faster R-CNN, Cascade R-CNN, DINO, RCS-YOLO, BGF-YOLO, CDC-YOLO, and the proposed CDCP-YOLO model. The metrics include precision, recall, mAP_0.5_, mAP_0.5:0.95_, number of parameters (Params), computational complexity (GFLOPs), and inference speed (FPS). Arrows indicate optimization direction: ↑ denotes that higher values are better (precision, recall, mAP_0.5_, mAP_0.5:0.95_, and FPS), while ↓ denotes that lower values are better (Params and GFLOPs). FPS, frames per second; GFLOPs, number of floating-point operations (×10⁹); mAP, mean average precision; mAP_0.5_, mAP at an intersection-over-union threshold of 0.5; mAP_0.5:0.95_, average mAP over several intersection-over-union thresholds (0.50-0.95); R-CNN, region-based convolutional neural network; YOLO, “you only look once” framework.

As seen in Figure 5, CDCP-YOLO was in the optimal or near-optimal state in terms of accuracy, model size, computational complexity, and inference speed, demonstrating the advantage of balancing accuracy and efficiency. It is particularly noteworthy that CDCP-YOLO significantly surpassed YOLOv5n (0.644 vs 0.601) and was superior to TOOD (0.644 vs 0.630) on mAP_0.5:0.95_, while it had a much lower parameter count and computational load than these models. This indicates that the model still has stable performance at high IoU thresholds, possessing good robustness and generalizability. CDCP-YOLO achieved high detection accuracy and inference speed under the premise of maintaining a small model size and low computational overhead.

### Ablation Experiment

Using YOLOv11n as a baseline, the effectiveness of each module is compared (Table 3).

**Table 3.** Ablation study results of individual modules on the Br35H brain tumor magnetic resonance imaging dataset, showing the incremental impact of the CSPP^a^, DCC^b^, CPCA^c^, and PHPS^d^ modules on detection performance and model efficiency.

Model	CSPP	DCC	CPCA	PHPS	Precision^e^	Recall	mAP_0.5^f^_	mAP_0.5:0.95^g^_	Params (M)^h^	GFLOPs^i^	FPS^j^
YOLOv11n	—^k^	—	—	—	0.904	0.853	0.918	0.585	2.58	6.3	142.2
C-YOLO	✓^l^	—	—	—	0.904	0.852	0.921	0.639	2.58	6.5	140
CD-YOLO	✓	✓	—	—	0.900	0.893	0.941	0.660	3.46	6.3	133
CDC-YOLO	✓	✓	✓	—	0.881	0.906	0.946	0.660	3.64	7.0	130
CDCP-YOLO	✓	✓	✓	✓	0.918	0.904	0.944	0.644	2.07	3.3	152

a CSPP: convolution Prewitt-and-pooling–based preprocessing.

b DCC: dynamic convolution–based C3k2.

c CPCA: channel prior convolutional attention.

d PHPS: progressive hybrid pruning strategy.

e Precision, recall, mAP_0.5_, and mAP_0.5:0.95_ were used to evaluate detection accuracy.

f mAP_0.5_: mean average precision at an intersection-over-union threshold of 0.5.

g mAP_0.5:0.95_: average mean average precision over several intersection-over-union thresholds (0.50-0.95).

h Params (M) was used to measure model size.

i GFLOPs: number of floating-point operations (×10⁹); used to measure computational complexity.

j FPS: frames per second; used to measure inference efficiency.

k Indicates that the corresponding module is absent in the model.

l A check mark (✓) indicates that the corresponding module is included in the model.

The mAP_0.5_ of the original YOLOv11n was 0.918, mAP_0.5:0.95_ was 0.585, parameter size was 2.58M, computational complexity was 6.3 GFLOPs, and inference speed was 142.2 FPS. After adding the CSPP module, the mAP_0.5_ of C-YOLO slightly increased to 0.921 and its mAP_0.5:0.95_ significantly increased to 0.639, while the number of parameters and computational complexity remained unchanged, indicating that the CSPP structure has a good effect on improving the model’s ability to capture fine-grained features. After introducing the DCC module, the detection accuracy was further improved: the mAP_0.5_ of CD-YOLO increased to 0.941, and the mAP_0.5:0.95_ increased to 0.660. Although the parameter size slightly increased to 3.46M, computational complexity remained at 6.3 GFLOPs, and inference speed dropped to 133 FPS, the model’s improvement in accuracy effectively enhanced its contextual modeling capability. Subsequently, with the addition of the CPCA module to obtain CDC-YOLO, the mAP_0.5_ reached 0.946 (the highest value), and the mAP_0.5:0.95_ remained unchanged at 0.660. Although parameter size slightly increased to 3.64M and inference speed dropped to 130 FPS, CDC-YOLO still performed outstandingly in terms of detection accuracy, further verifying the performance improvement effect of CPCA. On the basis of CDC-YOLO, PHPS was introduced to obtain CDCP-YOLO, which achieved a rebalance of model lightweighting and performance. This model reached 0.944 in mAP_0.5_ and 0.644 in mAP_0.5:0.95_, with only 2.07M parameters and computation complexity of 3.3 GFLOPs. The inference speed significantly increased to 152 FPS. This result shows that the PHPS basically retains high performance while compressing the model size.

**Figure 6.**
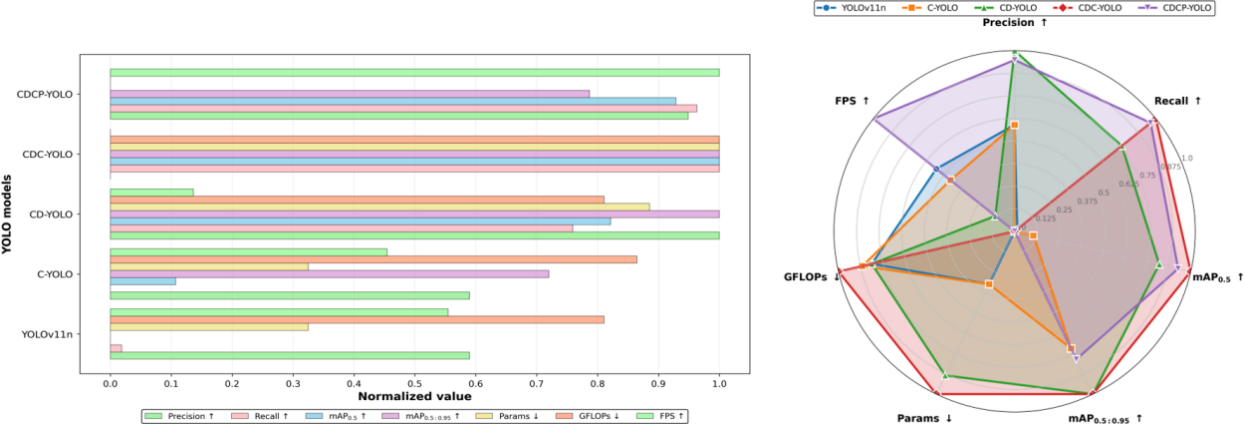
Ablation study results of the proposed CDCP-YOLO on the Br35H brain tumor magnetic resonance imaging dataset. The figure on the left shows the multimetric normalization bar chart, where all evaluation metrics are normalized to the range [0, 1] for fair comparison across models. The metrics include precision, recall, mAP_0.5_, mAP_0.5:0.95_, number of parameters (Params), computational complexity (GFLOPs), and inference speed (FPS). The figure on the right presents a radar-based performance analysis plot summarizing the overall accuracy-efficiency trade-off of different ablation variants (YOLOv11n, C-YOLO, CD-YOLO, CDC-YOLO, and CDCP-YOLO). Arrows indicate metric preference: ↑ denotes that higher values are better (precision, recall, mAP_0.5_, mAP_0.5:0.95_, and FPS), while ↓ denotes that lower values are better (Params and GFLOPs). This figure demonstrates how each module (convolution Prewitt-and-pooling–based preprocessing [CSPP], dynamic convolution–based C3k2 [DCC], channel prior convolutional attention [CPCA], and progressive hybrid pruning strategy [PHPS]) progressively improves detection accuracy while reducing model complexity. FPS, frames per second; GFLOPs, number of floating-point operations (×10⁹); mAP, mean average precision; mAP_0.5_, mAP at an intersection-over-union threshold of 0.5; mAP_0.5:0.95_, average mAP over several intersection-over-union thresholds (0.50-0.95); YOLO, “you only look once” framework.

As Figure 6 shows, CDCP-YOLO achieved a good balance in terms of accuracy, model size, computational load, and speed, showing a high degree of practicality and deployment advantages. Compared with other improved models, CDCP-YOLO had an absolute advantage in the 2 dimensions “fewest parameters” and “fastest inference,” while also maintaining excellent performance in detection accuracy.

### Performance on Different Datasets

To comprehensively evaluate the performance of CDCP-YOLO, the performance of YOLOv11, CDC-YOLO, and CDCP-YOLO was compared on different datasets (Table 4).

**Table 4.** Performance comparison of YOLOv11, CDC-YOLO, and CDCP-YOLO on 3 brain tumor magnetic resonance imaging datasets: Br35H, Roboflow, and Capstone.^a^

Dataset and model	Precision	Recall	mAP_0.5^b^_	mAP_0.5:0.95^c^_	Params (M)^d^	GFLOPs^e^	FPS^f^
Br35H							
YOLOv11	0.904	0.853	0.918	0.585	2.58	6.3	142.2
CDC-YOLO	0.881	0.906	0.946	0.660	3.64	7.0	130
CDCP-YOLO	0.918	0.904	0.944	0.644	2.07	3.3	152
Roboflow							
YOLOv11	0.615	0.467	0.561	0.276	2.58	6.3	117.6
CDC-YOLO	0.706	0.716	0.77	0.361	3.64	7.0	115
CDCP-YOLO	0.699	0.729	0.756	0.353	2.07	3.3	130.5
Capstone							
YOLOv11	0.768	0.806	0.840	0.567	2.58	6.3	132.2
CDC-YOLO	0.877	0.828	0.876	0.625	3.64	7.0	130.3
CDCP-YOLO	0.897	0.845	0.909	0.625	2.07	3.3	150

a The table reports detection accuracy metrics (precision, recall, mAP_0.5_, and mAP_0.5:0.95_) and efficiency metrics (Params, GFLOPs, and FPS) to evaluate both effectiveness and deployability across different datasets.

b mAP_0.5_: mean average precision at an intersection-over-union threshold of 0.5.

c mAP_0.5:0.95_: average mean average precision over several intersection-over-union thresholds (0.50-0.95).

d Params (M) was used to measure model size.

e GFLOPs: number of floating-point operations (×10⁹).

f FPS: frames per second.

On Br35H, CDCP-YOLO was superior to other models in terms of precision (0.918) and inference speed (152 FPS). Although its mAP_0.5_ was slightly lower than that of CDC-YOLO (0.944 vs 0.946), the outstanding performance of CDCP-YOLO in terms of parameter size (2.07M) and computational complexity (3.3 GFLOPs) makes it more suitable for practical application scenarios with limited resources. In addition, the mAP_0.5:0.95_ metric showed that it achieved a good trade-off between performance and computational efficiency. The Roboflow dataset represented a more challenging general-purpose detection scenario, resulting in lower overall detection accuracy than that achieved on the Br35H dataset. CDC-YOLO achieved the highest mAP_0.5_ (0.770) and mAP_0.5:0.95_ (0.361), while CDCP-YOLO performed best in recall (0.729), and it had a higher inference speed (130.5 FPS) than CDC-YOLO (115 FPS). The overall performance of YOLOv11 was the weakest, especially with obvious shortcomings in recall (0.467) and mAP_0.5:0.95_ (0.276), indicating that the original model lacked sufficient generalizability on this dataset. On Capstone, CDCP-YOLO once again demonstrated the best overall performance. It also showed the highest precision (0.897), mAP_0.5_ (0.909), and FPS (150), indicating that the model has stronger stability and efficiency in actual inference tasks. Although CDC-YOLO and CDCP-YOLO were comparable in mAP_0.5:0.95_ (both 0.625), CDCP-YOLO demonstrated higher comprehensive advantages by virtue of its smaller model size and faster speed.

**Figure 7.**
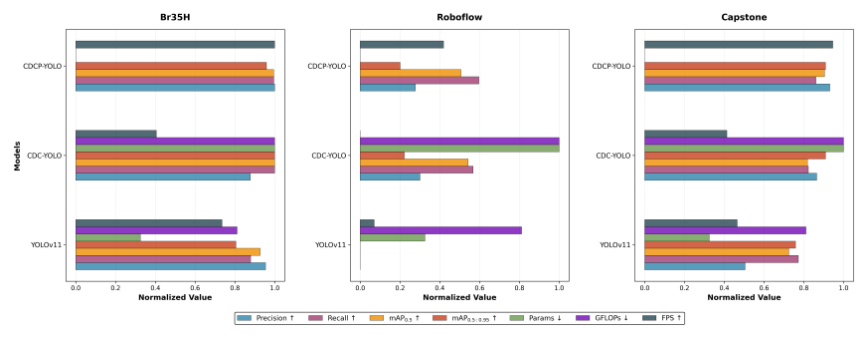
Multimetric normalization analysis of CDCP-YOLO and baseline models on 3 brain tumor magnetic resonance imaging datasets: Br35H, Roboflow and Capstone. Each graph corresponds to 1 dataset, showing the normalized performance of YOLOv11, CDC-YOLO, and CDCP-YOLO across multiple evaluation metrics. All metrics are normalized to the range [0, 1] to enable fair comparison across datasets with different scales. The evaluated metrics include precision, recall, mAP_0.5_, mAP_0.5:0.95_, number of parameters (Params), computational complexity (GFLOPs), and inference speed (FPS). Arrows indicate the optimization direction: ↑ denotes that higher values are better (precision, recall, mAP_0.5_, mAP_0.5:0.95_, and FPS), while ↓ denotes that lower values are better (Params and GFLOPs). FPS, frames per second; GFLOPs, number of floating-point operations (×10⁹); mAP, mean average precision; mAP_0.5_, mAP at an intersection-over-union threshold of 0.5; mAP_0.5:0.95_, average mAP over several intersection-over-union thresholds (0.50-0.95); YOLO, “you only look once” framework.

As seen in Figure 7, CDCP-YOLO performed outstandingly in terms of “fewest parameters,” “lowest computational load,” and “fastest FPS,” while also maintaining a leading position in detection accuracy metrics, demonstrating an extremely high balance and deployment advantage. YOLOv11, although fast in some scenarios, has lower precision and recall. CDC-YOLO had a slight advantage in precision, but its computational complexity and parameter size were higher.

### Impact of Different Attention Mechanisms

This paper introduces these modules including MixStructureBlock [42], MSCAttention [43], MSPABlock [44], and CPCA for comparing different attention mechanisms (Table 5).

**Table 5.** Comparative analysis of different attention mechanisms on the Br35H brain tumor magnetic resonance imaging dataset.^a^

Attention mechanism	Precision	Recall	mAP_0.5^b^_	mAP_0.5:0.95^c^_	Params (M)^e^	GFLOPs^d^	FPS^f^
+MixStructureBlock	0.827	0.665	0.754	0.354	67.90	11	120
+MSCAttention	0.891	0.877	0.933	0.624	2.00	3.3	117.6
+MSPABlock	0.883	0.863	0.924	0.637	2.50	3.7	110
+CPCA^g^	0.918	0.904	0.944	0.644	2.07	3.3	152

a The table reports detection accuracy metrics (precision, recall, mAP_0.5_, and mAP_0.5:0.95_) and efficiency metrics (Params, GFLOPs, and FPS) to evaluate the effectiveness and computational cost of different attention modules.

b mAP_0.5_: mean average precision at an intersection-over-union threshold of 0.5.

c mAP_0.5:0.95_: average mean average precision over several intersection-over-union thresholds (0.50-0.95).

d GFLOPs: number of floating-point operations (×10⁹).

e Params (M) was used to measure model size.

f FPS: frames per second.

g CPCA: channel prior convolutional attention.

**Figure 8.**
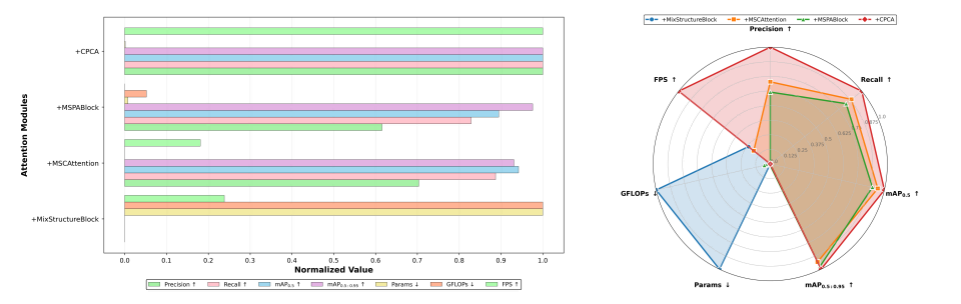
Comparative analysis of different attention mechanisms on the Br35H brain tumor magnetic resonance imaging dataset. The subfigure on the right shows the multimetric normalization bar chart, where all evaluation metrics are normalized to the range [0,1] for fair comparison among different attention modules. The compared attention mechanisms include MixStructureBlock, MSCAttention, MSPABlock, and the proposed channel prior convolutional attention (CPCA) module. The evaluated metrics include precision, recall, mAP_0.5_, mAP_0.5:0.95_, number of parameters (Params), computational complexity (GFLOPs), and inference speed (FPS). The subfigure on the right presents a radar-based performance analysis plot, summarizing the overall accuracy-efficiency trade-offs of different attention mechanisms. Arrows indicate metric preference: ↑ denotes that higher values are better (precision, recall, mAP_0.5_, mAP_0.5:0.95_, and FPS), while ↓ denotes that lower values are better (Params and GFLOPs). FPS, frames per second; GFLOPs, number of floating-point operations (×10⁹); mAP_0.5_, mean average precision at an intersection-over-union threshold of 0.5; mAP_0.5:0.95_, average mean average precision over several intersection-over-union thresholds (0.50-0.95).

As Figure 8 shows, CPCA performed excellently in terms of accuracy, efficiency, and resource consumption, making it the most cost-effective attention choice. In contrast, although MixStructureBlock has a complex structure, it was in a disadvantageous position in all performance indicators, indicating that the redundancy in its design was not converted into performance gains.

### Impact of Different Loss Functions

Comparative experiments were conducted by introducing loss functions such as GIoU [45], DIoU [46], EIoU [47], SIoU, ShapeIoU, PIoU, WIoU, and CIoU (Table 6).

**Table 6.** Performance comparison of different bounding box regression loss functions on the Br35H brain tumor magnetic resonance imaging dataset.^a^

Loss function	Precision	Recall	mAP_0.5^b^_	mAP_0.5:0.95^c^_	Params (M)^d^	GFLOPs^e^	FPS^f^
GIoU	0.893	0.820	0.926	0.635	2.07	3.3	150
DIoU	0.880	0.877	0.933	0.629	2.07	3.3	146.2
EIoU	0.867	0.787	0.875	0.546	2.07	3.3	126.1
SIoU	0.948	0.753	0.872	0.564	2.07	3.3	145
ShapeIoU	0.945	0.842	0.930	0.642	2.07	3.3	136.1
PIoU	0.890	0.885	0.923	0.646	2.07	3.3	124.8
WIoU	0.900	0.889	0.939	0.643	2.07	3.3	143
CIoU	0.899	0.873	0.944	0.644	2.07	3.3	152

a The table reports detection accuracy metrics (precision, recall, mAP_0.5_, and mAP_0.5:0.95_) and efficiency metrics (Params, GFLOPs, and FPS) to evaluate the impact of different loss functions on detection performance and computational efficiency.

b mAP_0.5_: mean average precision at an intersection-over-union threshold of 0.5.

c mAP_0.5:0.95_: average mean average precision over several intersection-over-union thresholds (0.50-0.95).

d Params (M) was used to measure model size.

e GFLOPs: number of floating-point operations (×10⁹).

f FPS: frames per second.

**Figure 9.**
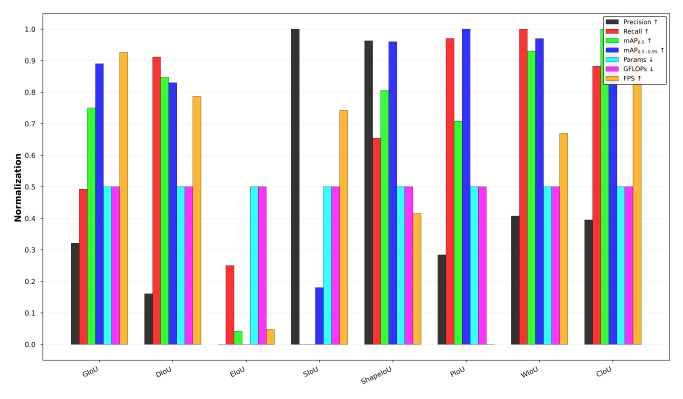
Multimetric normalization comparison of different bounding box regression loss functions on the Br35H brain tumor magnetic resonance imaging dataset. All evaluation metrics are normalized to the range [0,1] to enable fair comparison among different loss functions. The compared loss functions include GIoU, DIoU, EIoU, SIoU, ShapeIoU, PIoU, WIoU, and CIoU. The evaluated metrics include precision, recall, mAP_0.5_, mAP_0.5:0.95_, number of parameters (Params), computational complexity (GFLOPs), and inference speed (FPS). Arrows indicate optimization direction: ↑ denotes that higher values are better (precision, recall, mAP_0.5_, mAP_0.5:0.95_, and FPS), while ↓ denotes that lower values are better (Params and GFLOPs). FPS, frames per second; GFLOPs, number of floating-point operations (×10⁹); mAP, mean average precision; mAP_0.5_, mAP at an intersection-over-union threshold of 0.5; mAP_0.5:0.95_, average mAP over several intersection-over-union thresholds (0.50-0.95).

As Figure 9 shows, CIoU, as a new generation of bounding box regression loss function, significantly improved the accuracy and stability of target detection under the premise of ensuring model lightweighting, making it the most practical and valuable choice at present. At the same time, ShapeIoU and PIoU also had advantages in scenarios with high precision requirements, while WIoU provided good recall and generalization performance.

### Pruning Experiment

Five common pruning methods were systematically evaluated, including LAMP, L1, Random, GroupNorm, and PHPS (Table 7). All methods were kept consistent in terms of the number of parameters (2.07M) and computational complexity (3.3 GFLOPs) to facilitate a fair comparison of their performance differences.

**Table 7.** Performance comparison of different pruning strategies on the Br35H brain tumor magnetic resonance imaging dataset under the same parameter budget.^a^

Pruning method	Precision	Recall	mAP_0.5^b^_	mAP_0.5:0.95^c^_	Params^d^	GFLOPs^e^	FPS^f^
Lamp	0.858	0.891	0.941	0.640	2.07	3.3	137.1
L1	0.893	0.902	0.934	0.640	2.07	3.3	147
Random	0.866	0.902	0.940	0.637	2.07	3.3	150
GroupNorm	0.909	0.861	0.944	0.632	2.07	3.3	146.5
PHPS^g^	0.899	0.873	0.944	0.644	2.07	3.3	152

a The table reports detection accuracy metrics (precision, recall, mAP_0.5_, and mAP_0.5:0.95_) and efficiency metrics (Params, GFLOPs, and FPS) to evaluate the impact of different pruning methods on detection performance and inference efficiency.

b mAP_0.5_: mean average precision at an intersection-over-union threshold of 0.5.

c mAP_0.5:0.95_: average mean average precision over several intersection-over-union thresholds (0.50-0.95).

d Params (M) was used to measure model size.

e GFLOPs: number of floating-point operations (×10⁹).

f FPS: frames per second.

g PHPS: progressive hybrid pruning strategy.

The performance of PHPS was balanced and optimal across all performance indicators. The mAP_0.5_ was the same as the GroupNorm method at 0.944, but PHPS reached the highest value of 0.644 on mAP_0.5:0.95_ and also had the fastest inference speed (152 FPS), showing the best balance of precision and speed. GroupNorm had an advantage in precision (0.909), but recall rate was low (0.861), and mAP_0.5:0.95_ was not as good as that of PHPS, indicating that its strength in boundary fitting may affect the completeness of target detection. Among the other methods, L1 had a slight advantage in recall (0.902), and the overall precision was stable. Random pruning is simple and has not undergone structural optimization, but it performed well in terms of inference speed (150 FPS). LAMP was inferior in all indicators, especially precision (0.858) and mAP_0.5:0.95_ (0.640), indicating that its pruning method failed to effectively retain key features, weakening the model’s detection ability.

**Figure 10.**
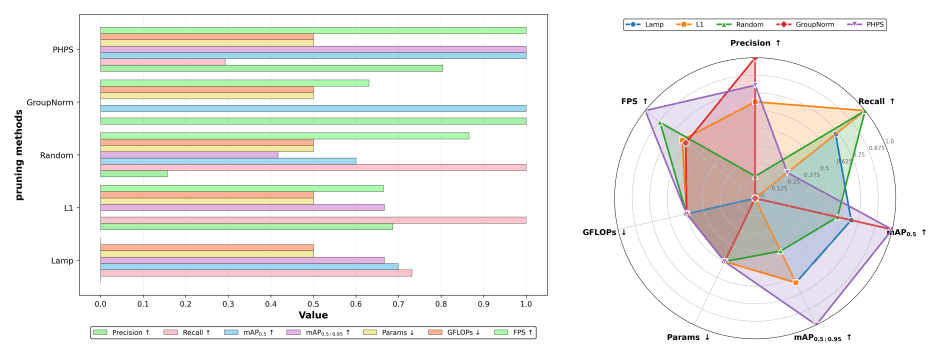
Comparative analysis of different pruning strategies on the Br35H brain tumor magnetic resonance imaging dataset under the same parameter budget. The left subfigure shows the multimetric normalization bar chart, where all evaluation metrics are normalized to the range [0,1] for fair comparison. The compared pruning methods include LAMP, L1-norm pruning, random pruning, GroupNorm-based pruning, and the proposed progressive hybrid pruning strategy (PHPS). The evaluated metrics include precision, recall, mAP_0.5_, mAP_0.5:0.95_, number of parameters (Params), computational complexity (GFLOPs), and inference speed (FPS). The right subfigure presents a radar-based performance analysis plot summarizing the overall accuracy-efficiency trade-offs of different pruning strategies. Arrows indicate optimization direction: ↑ denotes that higher values are better (precision, recall, mAP_0.5_, mAP_0.5:0.95_, and FPS), while ↓ denotes that lower values are better (Params and GFLOPs). FPS, frames per second; GFLOPs, number of floating-point operations (×10⁹); mAP, mean average precision; mAP_0.5_, mAP at an intersection-over-union threshold of 0.5; mAP_0.5:0.95_, average mAP over several intersection-over-union thresholds (0.50-0.95).

Figure 10 shows that PHPS was in the outermost layer in dimensions such as “precision,” “recall,” “mAP” and “inference speed,” representing that it scored the highest on each metric and had extremely strong practicality and deployment advantages. In contrast, the normalized evaluation of LAMP and GroupNorm showed obvious shortcomings, with deficiencies in detection accuracy and speed, respectively, making it difficult to meet the requirements of high-performance real-time tasks.

To assess robustness, we fixed the same fully trained baseline checkpoint and repeated the pruning procedure 5 times with different random seeds. For each run, we pruned the model to the same parameter budget as that reported in Table 7 and performed a single final fine-tuning stage. These results demonstrated that, although the mean mAP gap between PHPS and random pruning was limited, PHPS provided significantly improved robustness and stability, which is particularly important for reliable deployment in medical imaging applications (Table 8).

**Table 8.** Stability comparison of different pruning strategies under the same parameter budget on the Br35H brain tumor magnetic resonance imaging dataset.^a^

Pruning method	SD (mAP_0.5^b^_) ×10^-3^	SD (mAP_0.5:0.95^c^_) ×10^-3^
Lamp	3.35	5.66
L1	1.41	3.54
Random	7.07	2.12
GroupNorm	2.83	1.42
PHPS^d^	0.71	0.71

a The table reports the SD of mAP_0.5_ and mAP_0.5:0.95_ (scaled by 10⁻³) across multiple training runs, which reflects the robustness and training stability of each pruning method.

b mAP_0.5_: mean average precision at an intersection-over-union threshold of 0.5.

c mAP_0.5:0.95_: average mean average precision over several intersection-over-union thresholds (0.50-0.95).

d PHPS: progressive hybrid pruning strategy.

Although different pruning strategies vary in their effectiveness, they also differ in practical implementation costs. Unstructured pruning often introduces limited pruning time overhead but requires sparse inference support, whereas structured pruning incurs additional pruning and fine-tuning costs during training while enabling direct acceleration at inference. The proposed PHPS performs pruning offline during training and does not introduce extra computational overhead at inference, making it suitable for deployment without modifying existing inference pipelines.

### Five-Fold Cross-Validation

To further evaluate the statistical reliability of the performance gain between CDCP-YOLO and YOLOv11, we conducted a 5-fold cross-validation. The dataset was partitioned into 5 mutually exclusive folds. In each fold, 4 folds were used for training and the remaining one for testing, ensuring that each sample was evaluated exactly once.

All models were trained from scratch under identical training settings for each fold. The final performance was reported as the mean (SD) across the 5 folds.

The mean (SD) values of precision, recall, mAP_0.5_, and mAP_0.5:0.95_ across the 5 folds are reported in Table 9. The results showed that CDCP-YOLO consistently outperformed YOLOv11 across different folds, with lower variance and more stable performance, confirming that the observed improvement was statistically reliable and reproducible, rather than an artifact of random initialization.

**Table 9.** Five-fold cross-validation results of YOLOv11 and CDCP-YOLO on the Br35H brain tumor magnetic resonance imaging dataset.^a^

Model	Precision, mean (SD ×10^-3^)	Recall, mean (SD ×10^-3^)	mAP_0.5_, mean (SD ×10^-3^)	mAP_0.5:0.95_, mean (SD ×10^-3^)
YOLOv11	0.907 (17.880)	0.854 (2.492)	0.920 (3.030)	0.588 (11.500)
CDCP-YOLO	0.918 (1.410)	0.906 (2.610)	0.943 (2.590)	0.644 (1.410)

a The table reports the mean values of precision, recall, mAP_0.5_, and mAP_0.5:0.95_ across 5 folds, together with the corresponding SD (×10⁻³) values, which reflect the training stability and robustness of each model.

### Interpretability

**Figure 11.**
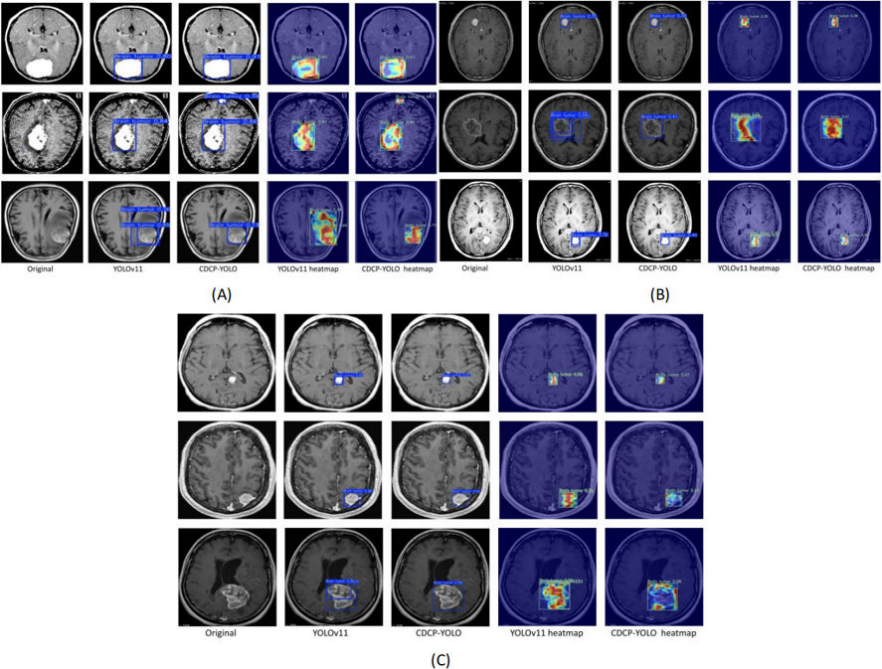
Qualitative visual comparison of detection and interpretability results between YOLOv11 and the proposed CDCP-YOLO model on 3 brain tumor magnetic resonance imaging (MRI) datasets. (A) Br35H dataset, (B) Roboflow dataset, and (C) Capstone dataset. For each dataset, the columns (from left to right) show the original MRI slice, detection results of YOLOv11, detection results of CDCP-YOLO, Eigen-CAM heat map of YOLOv11, and Eigen-CAM heat map of CDCP-YOLO. The heat map color gradient ranges from blue to red, indicating low to high activation intensity, respectively, where warmer colors correspond to regions that contribute most to the detection decision. Compared with YOLOv11, CDCP-YOLO produces more compact and accurate bounding boxes and generates more focused and lesion-aligned activation maps, especially for small, blurry, or irregular tumor regions. These results demonstrate the improved detection reliability and interpretability of the proposed framework. Eigen-CAM, Eigen-class activation mapping; YOLO, “you only look once” framework.

In Figure 11, for each dataset, the first column shows the original MRI image, and the remaining columns show the detection boxes generated by the YOLOv11 and CDCP-YOLO frameworks and the corresponding Eigen-CAM heat maps, respectively. The overall comparison results showed that CDCP-YOLO performed with higher detection confidence, more compact detection boxes, and more focused responses on different datasets, significantly outperforming the YOLOv11 baseline.

From both quantitative and qualitative perspectives: on Br35H, the detection confidence of CDCP-YOLO in the first image was much higher than YOLOv11; in the second image, YOLOv11 failed to detect the tumor on the left, while CDCP-YOLO successfully identified it; in the third image, YOLOv11 produced 2 bounding boxes with inaccurate positioning, while the detection box of CDCP-YOLO was closer to the lesion outline; the Eigen-CAM heat map also mainly covered the high-signal core and extended to the indistinct boundary. On Roboflow, the confidence level of CDCP-YOLO was also significantly higher than that of YOLOv11, and it was more accurate in terms of boundary fit and shape depiction. On Capstone, the 3 sets of images consistently showed that CDCP-YOLO not only has higher detection confidence but its bounding boxes were also more consistent with the actual lesion, and the thermal response was tighter and more focused. In MRI with complex backgrounds, YOLOv11 is often interfered with by shadows and anatomical structures (such as blood vessels and choroid plexus), leading to boundary offsets or missed detections. The heat map of CDCP-YOLO can stably focus on the tumor lesion area, effectively suppressing the interference of shadows and structural noise. This advantage stems from the deep integration of multiscale feature enhancement modules (CSPP and DCC) and CPCA, which significantly enhances the sensitivity and discrimination of tumor regions during feature extraction and judgment.

### Clinical Sensitivity and False Negative Analysis

In medical diagnosis, false negatives—particularly missed detections of small or visually ambiguous lesions—pose a critical risk. To evaluate the potential impact of pruning on clinical sensitivity, we further analyzed the behavior of the proposed PHPS from both quantitative and qualitative perspectives.

**Figure 12.**
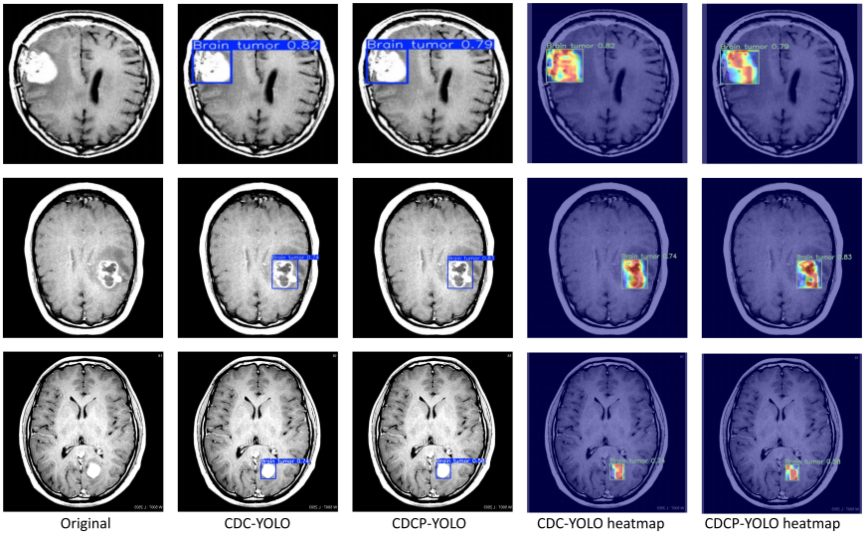
Qualitative comparison of detection sensitivity on small or ambiguous brain tumor regions before and after pruning on the Br35H brain tumor magnetic resonance imaging (MRI) dataset. From left to right, each column shows the original MRI slice, detection result of the unpruned CDC-YOLO, detection result of the pruned CDCP-YOLO, Eigen-CAM heat map of CDC-YOLO, and Eigen-CAM heat map of CDCP-YOLO. The heat map color gradient ranges from blue to red, indicating low to high activation intensity, respectively, where warmer colors correspond to regions that contribute most to the detection decision. Eigen-CAM, Eigen-class activation mapping; YOLO, “you only look once” framework.

Quantitatively, the recall remained stable across different pruning configurations, indicating that the progressive pruning process did not significantly impair the model’s ability to detect tumor regions. This suggests that the proposed strategy effectively preserved detection-critical channels during compression. Qualitatively, visualization results on representative cases involving small-scale or blurred tumor boundaries showed that the pruned model maintained consistent activation patterns compared with the unpruned baseline. These findings indicate that PHPS mitigates the risk of increased false negatives while achieving substantial model compression, thereby enhancing its suitability for clinical deployment. Despite significant model compression, the pruned CDCP-YOLO preserves consistent tumor localization and activation patterns compared with the unpruned baseline, especially for small-scale or low-contrast lesions. These results indicate that the proposed PHPS did not increase false negative rates and maintained clinical sensitivity under challenging conditions (Figure 12).

To statistically substantiate the “fine-grained” detection capability and robustness to small-scale tumors, we further evaluated the models using the AP_small_ metric, following the standard COCO evaluation protocol. As summarized in Table 10, CDCP-YOLO exhibited a superior sensitivity to small lesions compared to all baseline models. Specifically, CDCP-YOLO achieved an AP_small_ of 0.487, outperforming the YOLOv11n baseline (0.412) by a substantial margin of 18.2%. This quantitative improvement indicates that the integration of the CSPP and DCC modules for edge and feature enhancement, alongside the CPCA mechanism for precise spatial attention, effectively preserves the subtle morphological features of early-stage or small-scale tumors—details that are frequently overlooked by standard architectures. These results provide rigorous evidence that the proposed framework is not only efficient but also highly reliable for detecting clinically significant small lesions.

**Table 10.** Quantitative evaluation of small-lesion detection performance, AP_small_ (the baseline YOLOv11n and the proposed CDCP-YOLO model), on the Br35H brain tumor magnetic resonance imaging dataset using the standard Common Objects in Context (COCO)–style detection metrics.

Model	mAP_0.5^a^_	AP_small^b^_	Relative gain
YOLOv11n	0.918	0.412	—^c^
CDCP-YOLO	0.944	0.487	+18.2%

a mAP_0.5_ denotes the mean average precision at an intersection-over-union threshold of 0.5.

b AP_small_ denotes the average precision computed only for small objects, following the COCO definition (ie, objects whose bounding-box area falls within the “small” size range specified by the COCO evaluation protocol).

c Not applicable.

**Figure 13.**
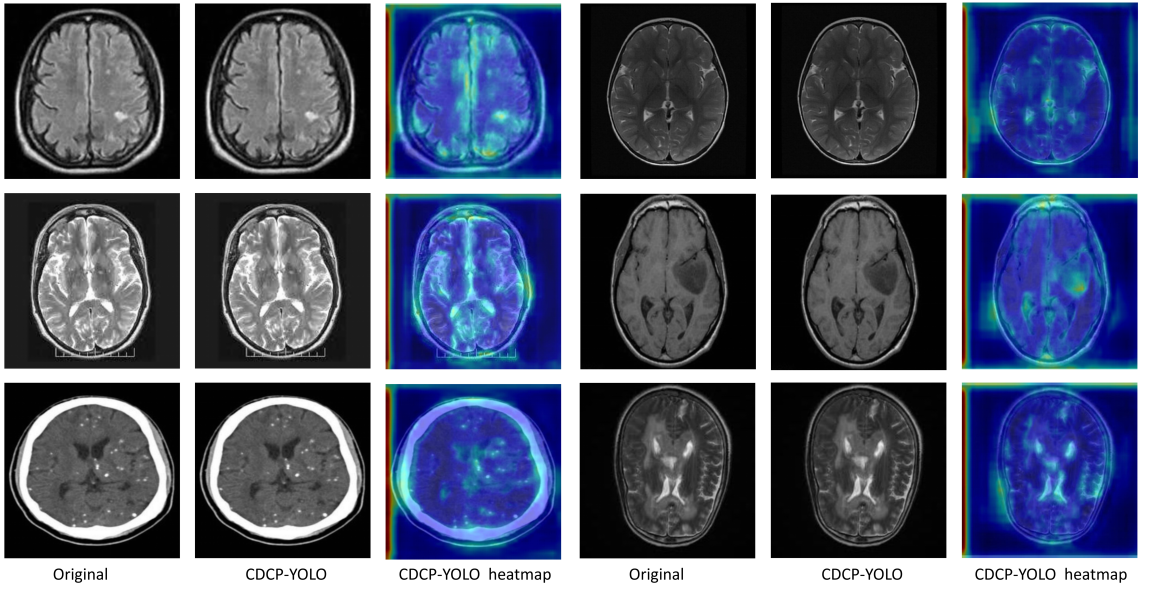
Representative examples of negative-control validation on healthy brain magnetic resonance imaging (MRI) slices using inference-only CDCP-YOLO and Eigen-CAM visualization. This figure presents an inference-only negative control experiment conducted on healthy brain MRI slices without tumor lesions, used to evaluate the specificity and safety of the proposed CDCP-YOLO model. The healthy slices were obtained from a publicly available brain MRI dataset and were not included in the training process. For each example, the first column shows the original MRI slice, the second column shows the detection result produced by CDCP-YOLO, and the third column presents the corresponding Eigen-CAM heat map overlaid on the original image. The color scale of the heat map represents the relative contribution of image regions to the model’s prediction, where warm colors (red/yellow) indicate higher activation intensity, and cool colors (blue) indicate low or background-level responses. Eigen-CAM, Eigen-class activation mapping; YOLO, “you only look once” framework.

To further assess the reliability and specificity of CDCP-YOLO, we conducted an inference-only validation on a negative control cohort of 50 healthy brain MRI slices obtained from the brain tumor dataset [48]. This test aimed to verify that the model does not produce “hallucinated” detections on normal anatomical structures. As shown in Figure 13, the model successfully classified all healthy slices as negative, producing no false positive detection boxes. Moreover, the Eigen-CAM visualizations showed only very weak background-level activations (cool colors) within healthy brain regions, without the high-intensity highlighted responses typically observed in tumor areas. This negative control experiment provided empirical evidence for the model’s robust safety profile, ensuring that its high tumor sensitivity is not achieved at the expense of misdiagnosing healthy anatomy.

### Hyperparameter Sensitivity Analysis of PHPS

To further assess the robustness of the proposed method, we conducted a sensitivity analysis on key training and pruning hyperparameters involved in PHPS. Specifically, we analyzed the sparsity coefficient (SC) and the learning rate (LR) schedule, as these parameters directly influence pruning behavior and convergence stability.

In this analysis, only one hyperparameter was varied at a time while all other settings were kept fixed, allowing us to isolate the effect of each parameter. The results are summarized in Table 11. As shown, the proposed method exhibited stable detection performance across a reasonable range of SC values, indicating that PHPS is not overly sensitive to the pruning threshold. In addition, different learning rate schedules led to comparable performance, suggesting that the training process remained robust under different optimization dynamics.

**Table 11.** Sensitivity analysis of key hyperparameters in the progressive hybrid pruning strategy (PHPS) on the Br35H brain tumor magnetic resonance imaging dataset.^a^

Hyperparameter	Setting	Precision	Recall	mAP_0.5^b^_	mAP_0.5:0.95^c^_
SC^d^	0.2	0.912	0.898	0.939	0.637
	0.3	0.918	0.904	0.944	0.644
	0.4	0.910	0.895	0.936	0.631
LR^e^ schedule	StepLR	0.914	0.899	0.941	0.639
	CosineAnnealingLR	0.918	0.904	0.944	0.644
	CosineAnnealingWarmRestarts	0.916	0.902	0.940	0.635

a The table reports the detection performance under different settings of the sparsity coefficient (SC) and learning rate (LR) scheduling strategies, including StepLR, CosineAnnealingLR, and CosineAnnealingWarmRestarts. Detection accuracy was evaluated using precision, recall, mAP_0.5_ and mAP_0.5:0.95_.

b mAP_0.5_: mean average precision at an intersection-over-union threshold of 0.5.

c mAP_0.5:0.95_: average mean average precision over several intersection-over-union thresholds (0.50-0.95).

d SC: sparsity coefficient.

e LR: learning rate.

### Ablation Experiments for Architectural and Pruning Analysis

To rigorously isolate the architectural contributions of the proposed CSPP, DCC, and CPCA modules and analyze the impact of pruning, a pruned baseline model (YOLOv11n-1) was included in the ablation study, as summarized in Table 12. Specifically, the standard YOLOv11n backbone was pruned to the same parameter budget (2.07M) using the same PHPS and fine-tuning protocol but without introducing any of the proposed architectural modules. This design allows us to decouple the effect of pruning from that of architectural enhancement. As shown in Table 12, applying PHPS to the standard YOLOv11n resulted in performance degradation, with mAP_0.5_ dropping from 0.918 to 0.902. This outcome demonstrates that aggressive pruning on a baseline backbone without specialized modules inevitably disrupts critical feature pathways, challenging the notion that pruning alone can enhance model capability. In contrast, the proposed CDCP-YOLO model achieved a significantly higher mAP_0.5_ of 0.944 under the same 2.07M parameter budget. These results clarify that the observed performance gains originated from the architectural enhancements—CSPP, DCC, and CPCA—which provided robust feature representation that successfully compensated for the pruning-induced losses. This confirms that jointly designing architectural enhancements and progressive pruning is essential for achieving a superior accuracy-efficiency trade-off in resource-constrained environments.

**Table 12.** Ablation study isolating the contributions of architectural modules and pruning, through comparison of (1) the unpruned baseline YOLOv11n; (2) a pruned baseline (YOLOv11n-1) obtained by applying the progressive hybrid pruning strategy (PHPS) to the standard YOLOv11n (without CSPP^a^, DCC^b^, or CPCA^c^) to match the parameter budget of 2.07M; and (3) the proposed CDCP-YOLO model, which integrated the proposed architectural modules (CSPP, DCC, and CPCA) and was pruned and fine-tuned under the same PHPS setting.

Model	Precision	Recall	mAP_0.5^d^_	mAP_0.5:0.95^e^_	Params (M)^f^	GFLOPs^g^	FPS^h^
YOLOv11n	0.904	0.853	0.918	0.585	2.58	6.3	142.2
YOLOv11n-1	0.905	0.855	0.902	0.580	2.07	2.8	145.3
CDCP-YOLO	0.918	0.904	0.944	0.644	2.07	3.3	152

a CSPP: convolution Prewitt-and-pooling–based preprocessing.

b DCC: dynamic convolution–based C3k2.

c CPCA: channel prior convolutional attention.

d mAP_0.5_: mean average precision at an intersection-over-union threshold of 0.5.

e mAP_0.5:0.95_: average mean average precision over several intersection-over-union thresholds (0.50-0.95).

f Params (M) was used to measure model size.

g GFLOPs: number of floating-point operations (×10⁹).

h FPS: frames per second.

### Impact of Training Budget on Performance Gain

To verify that the performance gains were not merely a result of longer total training duration, we retrained the YOLOv11n baseline for 600 epochs (YOLOv11n-2), matching the combined 2-stage budget of CDCP-YOLO. Results demonstrated that despite doubling the convergence time, the mAP_0.5_ of YOLOv11n only achieved a negligible increase of 0.001, remaining significantly below the value (0.944) achieved by CDCP-YOLO (Table 13). This finding underscored that the core contributions to detection accuracy stemmed from the proposed feature enhancement modules and the PHPS strategy rather than extended training time.

**Table 13.** Impact of training budget alignment on model performance on the Br35H brain tumor magnetic resonance imaging dataset, assessed through comparison of the detection performance under three experimental settings: (1) the standard YOLOv11n baseline trained for 300 epochs, (2) the YOLOv11n baseline retrained for 600 epochs to align with the total 2-stage training budget of CDCP-YOLO (denoted as YOLOv11n-2), and (3) the proposed CDCP-YOLO trained using a 2-stage training strategy (300 epochs of initial training followed by 300 epochs of postpruning fine-tuning).

Model	Total epochs, n	Precision	Recall	mAP_0.5^a^_	mAP_0.5:0.95^b^_	Params (M)^c^	GFLOPs^d^
YOLOv11n	300	0.904	0.853	0.918	0.585	2.58	6.3
YOLOv11n-2	600	0.905	0.855	0.919	0.580	2.58	6.3
CDCP-YOLO	600	0.918	0.904	0.944	0.644	2.07	3.3

a mAP_0.5_: mean average precision at an intersection-over-union threshold of 0.5.

b mAP_0.5:0.95_: average mean average precision over several intersection-over-union thresholds (0.50-0.95).

c Params (M) was used to measure model size.

d GFLOPs: number of floating-point operations (×10⁹).

### Computational Overhead and Deployment Considerations

Although different pruning strategies vary in their compression effectiveness, they also differ in practical implementation cost. The computational overhead of pruning methods can be analyzed from 3 aspects: pruning time overhead, fine-tuning cost, and inference-time efficiency.

Unstructured pruning typically introduces limited overhead during pruning but requires specialized sparse inference support at deployment. In contrast, structured pruning methods incur additional pruning and fine-tuning costs during training while enabling direct reductions in parameter size and number of floating-point operations (FLOPs) without modifying standard inference pipelines.

The proposed strategy PHPS performs pruning offline during training in a stage-wise manner. Consistent with the standard characteristics of structured pruning, PHPS physically removes redundant parameters from the network, resulting in a reduced model size that naturally avoids additional computational overhead during the inference phase. This lack of inference overhead is a direct consequence of the physical parameter removal inherent to structured pruning rather than a unique architectural capability of the PHPS itself.

This design makes PHPS particularly suitable for real-world deployment scenarios, such as edge devices or clinical systems, where inference efficiency and implementation simplicity are critical and modifying inference engines is impractical.

## Discussion

### Principal Findings

This study proposes CDCP-YOLO, an interpretable and lightweight brain tumor MRI detection framework that integrates feature enhancement, channel-prior attention, and progressive hybrid pruning. Experimental results across 3 public datasets demonstrated that CDCP-YOLO achieved a favorable balance between detection accuracy, computational efficiency, and model interpretability for slice-level brain tumor localization tasks. Notably, the proposed method consistently outperformed the YOLOv11 baseline while reducing parameters and GFLOPs by nearly half.

Furthermore, 5-fold cross-validation confirmed that the observed performance gains were statistically stable rather than artifacts of random initialization. The integration of Eigen-CAM enables visually consistent and task-aligned explanations that highlight tumor regions across MRI slices.

### Strengths and Limitations

Compared with prior YOLO-based brain tumor detectors that primarily rely on deeper architectures, additional detection heads, or external pretraining, CDCP-YOLO adopts a task-driven co-design strategy. Unlike RCS-YOLO or BGF-YOLO, which emphasize reparameterization or feature fusion, the proposed method enhances edge-sensitive feature extraction at the input stage and introduces dynamic convolution to adapt to heterogeneous tumor morphologies. In contrast to PK-YOLO, which depends on external pretrained knowledge, CDCP-YOLO improves representation capacity through internal data-adaptive mechanisms, making it more suitable for limited or domain-specific medical datasets. Moreover, most existing pruning-based detectors use one-shot or single-criterion pruning, whereas the proposed PHPS method preserves detection-critical structures through progressive and structure-aware pruning.

Several limitations should be acknowledged. First, the Br35H dataset used in this study does not provide patient identifiers, and the train, validation, and test split is therefore performed at the image (slice) level following the official dataset partitioning. As a result, strict patient-level splitting cannot be enforced, which may limit the assessment of patient-level generalization. This limitation is inherent to the dataset itself and will be addressed in future work using datasets with explicit patient-level annotations. Second, all datasets used in this study (Br35H, Roboflow, and Capstone) consist exclusively of tumor-positive MRI slices and do not include healthy control images. Consequently, strict negative control visualization for CAM-based interpretability cannot be conducted without introducing external data. Future work will incorporate MRI cohorts with healthy subjects to enable negative control interpretability analysis and more rigorous validation of model behavior on normal anatomy. Third, while the proposed pruning strategy preserves recall performance, extremely small or highly ambiguous lesions may still pose challenges under aggressive compression. This limitation was partially addressed through qualitative sensitivity analysis, but further prospective validation is required. Finally, this study focused on 2D slice–based detection and did not explicitly model interslice spatial continuity, which could further improve robustness in volumetric MRI analysis.

### Future Directions

Future work will focus on expanding the dataset through multi-institutional collaboration and evaluating the framework on volumetric MRI sequences. In addition, integrating efficient transformer–based modules within the 2-stage framework will be explored to further enhance contextual modeling while maintaining deployability. Clinical-oriented studies, including cost-benefit and workflow integration analyses, will also be conducted to support real-world adoption.

### Conclusions

In conclusion, this study demonstrated that CDCP-YOLO achieves a well-balanced integration of detection accuracy, computational efficiency, and interpretability for slice-level brain tumor localization in MRI images. The proposed framework is built upon a task-driven co-design, incorporating structure-aware feature enhancement, dynamic convolution, and a channel-prior attention mechanism to strengthen discriminative feature representation. In particular, the attention module guides the network to focus on lesion-relevant tumor regions, improving robustness to small lesions and tumors with ambiguous boundaries.

Furthermore, the proposed strategy PHPS effectively compresses the model by jointly considering global sparsity and local structural dependency, enabling substantial reductions in parameters and computational cost while preserving detection-critical pathways. In combination with the integrated Eigen-CAM–based interpretability design, CDCP-YOLO provides visually consistent and task-aligned explanations that reflect the model’s detection behavior, enhancing the transparency and reliability of the framework.

Overall, the experimental results confirmed the feasibility of deploying lightweight, attention-enhanced, and interpretable object detection models for efficient slice-level brain tumor analysis and screening in MRI images. This work provides a practical and reliable solution for resource-constrained imaging environments and represents a meaningful step toward methodological advancement and potential clinical translation, rather than a direct clinical diagnostic system.

## Abbreviations

CA: channel attention
CAM: class activation mapping
CNN: convolutional neural network
CPCA: channel prior convolutional attention
CSPP: convolution Prewitt-and-pooling–based preprocessing
DCC: dynamic convolution–based C3k2
DCM: disease classification module
FLOPs: number of floating-point operations
FPS: frames per second
GFLOPs: number of floating-point operations (×10⁹)
Grad-CAM: gradient-weighted class activation mapping
HSV: hue-saturation-value
IoU: intersection over union
IQM: image quality module
mAP: mean average precision
MRI: magnetic resonance imaging
PHPS: progressive hybrid pruning strategy
R-CNN: region-based convolutional neural network
RCS: reparameterized convolution combined with channel shuffle
SA: spatial attention
SGD: stochastic gradient descent
SSD: single shot multibox detector
YOLO: you only look once
